# A fluorescent activatable probe for imaging intracellular Mg^2+^ †
†Electronic supplementary information (ESI) available: Additional synthetic details and characterisation, spectral and imaging data. See DOI: 10.1039/c7ob02965a


**DOI:** 10.1039/c7ob02965a

**Published:** 2017-12-19

**Authors:** Ryan Treadwell, Fabio de Moliner, Ramon Subiros-Funosas, Toby Hurd, Kirsten Knox, Marc Vendrell

**Affiliations:** a Medical Research Council Centre for Inflammation Research , The University of Edinburgh , EH16 4TJ Edinburgh , UK . Email: marc.vendrell@ed.ac.uk; b MRC Human Genetics Unit , Institute of Genetics and Molecular Medicine , The University of Edinburgh , EH4 2XU Edinburgh , UK; c Institute of Molecular Plant Sciences , The University of Edinburgh , EH9 3BF Edinburgh , UK

## Abstract

An activatable BODIPY probe for *in vitro* detection and fluorescence cell imaging of free Mg^2+^ without interference from Ca^2+^ is described. Fluorescence amplification of the probe is observed upon detection of physiological concentrations of Mg^2+^ due to reduced rotation of the fluorophore and effective chelation by a quinolizine-based core.

## Abstract 2

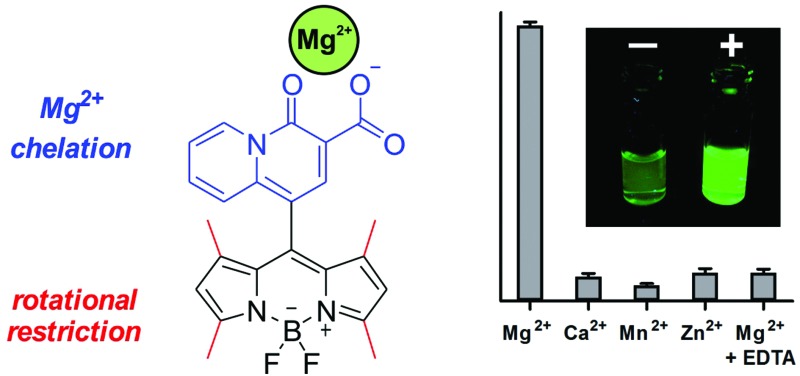
An BODIPY probe for detection and imaging of Mg^2+^ without interference from Ca^2+^ is described.

## Introduction

Intracellular magnesium (Mg^2+^) plays a crucial role in the physiological activity of live cells. At concentrations below 30 mM, Mg^2+^ is known to regulate and participate in a range of fundamental biological processes, including metabolic pathways, DNA synthesis and being a co-factor for more than 600 enzymes. Consequently, the concentration of free unbound intracellular Mg^2+^ falls to around 0.5–1.2 mM.^1^ A number of pathological disorders have been correlated to abnormal levels of Mg^2+^ within the body.^2^ For instance, low levels of Mg^2+^ are associated with the progression of Parkinson's disease, characterised by a depletion in the number of dopaminergic neurons in the brain.^3^ Therefore, the importance of Mg^2+^ in multiple physiological conditions demands new chemical tools to investigate the storage, concentration and homeostasis of Mg^2+^ in both health and disease.

Small organic fluorophores offer a cheap, reliable and non-disruptive method of cellular analysis, and have been widely reported to detect a plethora of biomolecules and enzymes, as well as to monitor biological processes and discriminate cell types.^4^–^9^ Advances in fluorophore development have led to the generation of smart or activatable probes, which are chemically designed so that their photophysical properties depend on an external stimulus. The fine-tuning of these chemical architectures not only allows spatiotemporal control of the readout of the probes but also provides enhanced signal-to-noise ratios, which boost their sensitivity in imaging studies under fluorescence microscopy. Furthermore, when significant differences are achieved between the readouts of activated and non-activated fluorophores, quantitative measurements of specific analytes or biological events can be performed. In the context of divalent cation sensing, since the pioneering work from Tsien *et al.*,^10^ multiple fluorescent probes have been reported for *in vivo* and *in vitro* tracking of divalent cations.^11^–^13^ Numerous chemical probes have been developed to monitor the levels of Ca^2+^, the most abundant cation, with only a modest number of probes being available for sensing Mg^2+^. A major challenge in the development of Mg^2+^-responding probes is to ensure that the fluorescence signal is elicited only in response to Mg^2+^ and no other divalent intracellular cations, with Ca^2+^ being a major interference due to its high intracellular levels. Mag-indo-1 and Mag-fura-2 are commercially available probes based on the *O*-aminophenol-*N*,*N*,*O*-triacetic acid (APTRA) chelating group, which can strongly bind to Mg^2+^ as well as to other intracellular divalent cations.^14^ Remarkable work has been also recently undertaken by the group of Buccella with the adaptation of APTRA to a range of chemical scaffolds (*e.g.* HaloTag-binding molecules, ratiometric and red-emitting fluorophores) to improve its capabilities as imaging reagents.^15^–^17^ Other chelating groups have been reported for enhanced recognition of Mg^2+^ and minimal cross-reactivity with Ca^2+^. Among these, the 4-oxo-4*H*-quinolizine-3-carboxylic acid scaffold reported by Levy *et al.*^18^ offers good discrimination between the two major divalent ions and can be effectively conjugated to fluorophores (*e.g.* fluorescein) to produce probes that respond to Mg^2+^ chelation by photoinduced electron transfer (PeT).^19^ However, the limited cell permeability of fluorescein hinders the use of these dyes for visualising intracellular Mg^2+^ levels in live cells. In addition to high cell permeability, the 4,4-difluoro-4-bora-3a,4a-diaza-*s*-indacene (BODIPY) scaffold presents excellent photophysical features for live cell imaging,^20^–^25^ such as narrow excitation and emission and excellent photostability. Furthermore, the fluorescence emission of BODIPY can be fine-tuned *via* incorporation of chelating groups at the *meso* position, which affect intramolecular PeT and the overall emission of the fluorophores.^26^–^29^ Therefore, we envisioned that the introduction of the 4-oxo-4*H*-quinolizine-3-carboxylic acid into the cell-permeable BODIPY scaffold might render novel activatable fluorophores for imaging intracellular levels of Mg^2+^.

## Results and discussion

### Rational design and chemical synthesis of Mg^2+^ probes

**Fig. 1:**
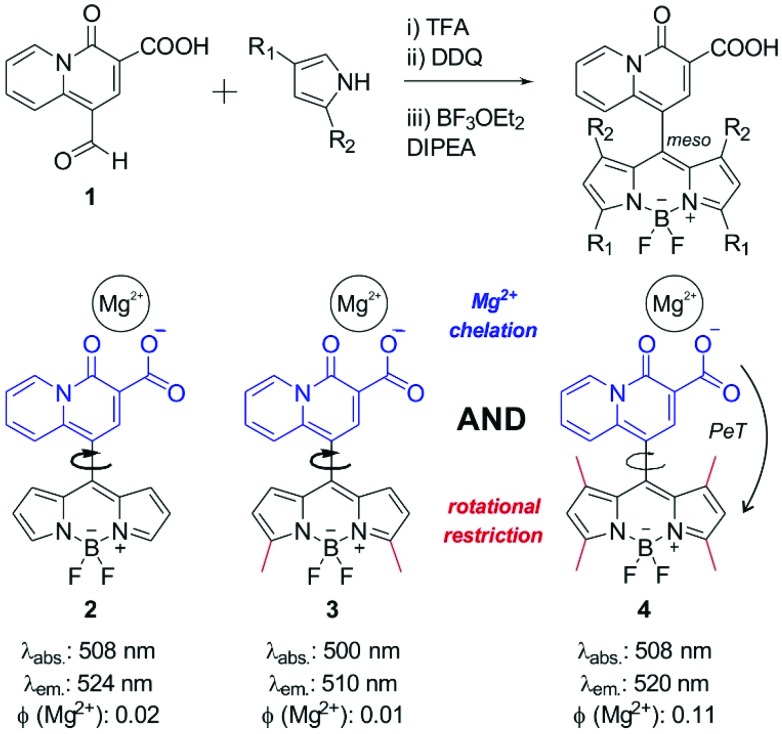
Simplified synthetic scheme for the preparation of Mg-sensitive BODIPY fluorophores based on the 4-oxo-*4H*-quinolizine-3-carboxylic acid chelating scaffold. Below are represented the chemical structures of BODIPY fluorophores **2**, **3** and **4** and their corresponding activation modes upon Mg^2+^ recognition. Fluorescence quantum yields were determined relative to fluorescein in basic ethanol.

First, we synthesized the quinolizine chelating moiety **1** (Fig. 1), which incorporates a β-diketone moiety to selectively chelate the Mg^2+^ ion and an aldehyde functional group for adaptation to the synthesis of symmetric BODIPY fluorophores.^30^ The 4-oxo-4*H*-quinolizine-3-carboxylic acid moiety shows enhanced selectivity for Mg^2+^ over Ca^2+^ when compared to other chelating groups (*e.g.* APTRA). The synthesis of the BODIPY fluorophore **2** was achieved in a one-pot single reaction by conjugation of the aldehyde **1** to pyrrole using slightly modified reported procedures^31^ to isolate the BODIPY compound **2** (Fig. 1) and examine its properties as a Mg^2+^-sensitive fluorophore.

The low fluorescence quantum yield (*i.e.* around 2%) of compound **2** after incubation with Mg^2+^ proved ineffective for the detection of Mg^2+^ levels within the physiological range. Such weak fluorescence emission can be explained due to the free rotation of the quinolizine group conjugated to the *meso* position, which might result in the energy of the excited state being lost as rotational energy rather than emitted as fluorescence. We assessed this hypothesis by examining the fluorescence emission of compound **2** in environments of increasing viscosity (Fig. S1 in ESI†). The enhancement of fluorescence intensity in glycerol-containing media confirmed the free rotation of the quinolizine group around the *meso* position as a major cause for the low fluorescence quantum yield of compound **2**.

We envisioned that BODIPY probes where the fluorescence enhancements derived from Mg^2+^ chelation and restricted rotation were combined in a single molecular framework might display suitable fluorescent quantum yields for Mg^2+^ detection in cells. As such, we designed and synthesized two additional BODIPY compounds with two or four methyl substituents (**3** and **4** respectively, Fig. 1). Whereas compound **3** would retain similar rotational freedom to compound **2**, the steric hindrance around the quinolizine core in compound **4** would minimise its free rotation. Compounds **3** and **4** were synthesized by condensation of the aldehyde **1** to 2-methylpyrrole or 2,4-dimethylpyrrole respectively, and isolated with reasonable synthetic yields. The excitation and emission wavelengths of all quinolizine-BODIPY derivatives (**2–4**) are similar (Fig. 1 and Fig. S2 in ESI†) as well as their photostability properties (Fig. S3 in ESI†). However, their fluorescence quantum yields were significantly different, with the tetramethyl-substituted BODIPY **4** showing the highest fluorescence quantum yield after binding to Mg^2+^ (11%, Fig. 1 and Fig. S4 in ESI†). This observation supports our rational design of BODIPY probes combining the chelating 4-oxo-4*H*-quinolizine-3-carboxylic acid with restricted rotational features to maximise the fluorescence response upon Mg^2+^ recognition.

### *In vitro* spectral characterisation of Mg^2+^ binding

**Fig. 2:**
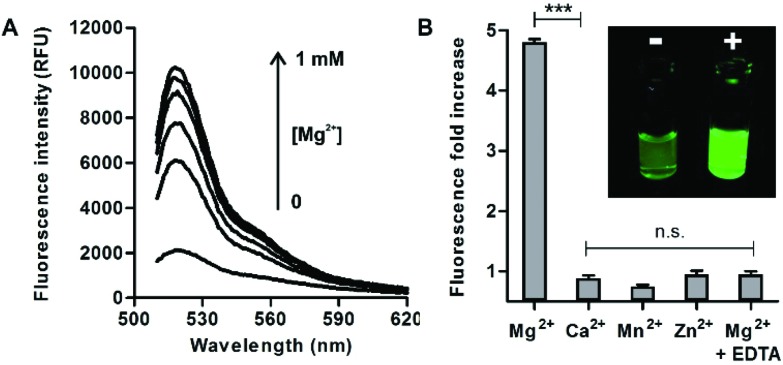
(A) Fluorescence emission of compound **4** (10 μM, H_2_O : EtOH 1 : 1) upon incubation with increasing concentrations (0, 0.1, 0.25, 0.5, 0.75 and 1 mM) of Mg^2+^; *λ*_exc._: 450 nm. (B) Comparative fluorescence of compound **4** upon incubation with different divalent cations at their respective physiological concentrations (Mg^2+^, Ca^2+^, Mn^2+^: 1 mM; Zn^2+^: 1 μM; EDTA: 5 mM). Inset: Pictograms of compound **4** under 365 nm excitation in solutions without (left) or with Mg^2+^ (right). *** for *p* < 0.001, n.s. for *p* > 0.05.

In view of the spectral properties of compound **4**, we examined its application for the detection and visualisation of intracellular Mg^2+^ levels. As shown in Fig. 2A, compound **4** showed a dose-dependent fluorescence increase upon incubation with concentrations of Mg^2+^ within its physiological range (*i.e.* up to 1 mM). Furthermore, compound **4** showed excellent discrimination of Mg^2+^*vs*. other divalent cations, with around 4-fold selectivity over Ca^2+^-the most abundant divalent cation found in cells and tissues- and marginal cross-reactivity with Mn^2+^ and Zn^2+^ at their physiological concentrations (Fig. 2B and Fig. S5 in ESI†).^32^,^33^ Mn^2+^ is present in cells in a relatively broad range of concentrations (*i.e.* between 0.04 and 2 mM)^34^ whereas Zn^2+^ is predominantly bound as a co-factor in protein complexes^35^ and found as a free cation in cells at very low concentrations (*i.e.* in the pM range). Notably, the co-incubation with Mg^2+^ and the chelating agent ethylenediaminetetraacetic acid (EDTA) did not lead to any fluorescence enhancement (Fig. 2B). This result confirms that, together with the lack of rotational energy loss, the blockage of the intramolecular PeT due to Mg^2+^ chelation is likely to be the main mechanism responsible for the fluorescence response of compound **4**, as reported for similar chelator-based fluorogenic probes.^36^–^38^ The fluorescence increase of compound **4** upon chelation of Mg^2+^ was readily detected by the naked eye, as an indication of its high sensitivity with a limit of detection in the low micromolar range (2 μM, Fig. S6 in ESI†).

**Fig. 3:**
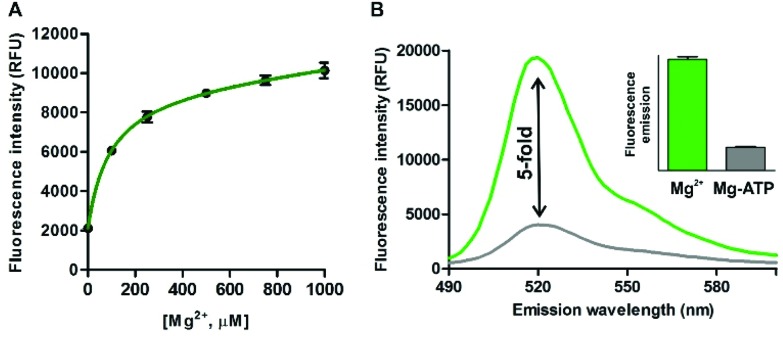
(A) Determination of the dissociation constant of the Mg^2+^-**4** adduct by measuring the fluorescence intensity of compound **4** (10 μM, H_2_O : EtOH 1 : 1) at increasing concentrations of Mg^2+^. Values represented as means ± s.e.m. (*n* = 3). *K*_d_: 92 ± 30 μM. (B) Emission spectra of compound **4** (50 μM) upon incubation with 2 mM free Mg^2+^ (green) and 2 mM Mg-ATP (grey); *λ*_exc._: 450 nm. (Inset) Quantification of the fluorescence intensities for both conditions. Values represented as means ± s.d. (*n* = 3).

Further detailed characterisation studies showed that the Mg^2+^-**4** adduct presented a *K*_d_ value of around 100 μM (Fig. 3A), in the range of DCHQ-based quinoline fluorophores^39^ and an order of magnitude lower than APTRA-based Mg^2+^-responding fluorophores.^16^ We also analysed the fluorescence response of the Mg^2+^-**4** adduct to different pH values and observed no major dependence on pH (Fig. S7 in ESI†). Finally, we compared the fluorescence response of compound **4** to both free Mg^2+^ as well as Mg-ATP, which is found in relatively high concentrations inside metabolically active cells.^40^ As shown in Fig. 3B, compound **4** showed around 5-fold brighter fluorescence emission when bound to free Mg^2+^ in comparison to the same concentration of Mg-ATP, asserting its utility to monitor changes in the levels of free Mg^2+^ within the intracellular physiological range.

### Fluorescence cell and tissue imaging

**Fig. 4:**
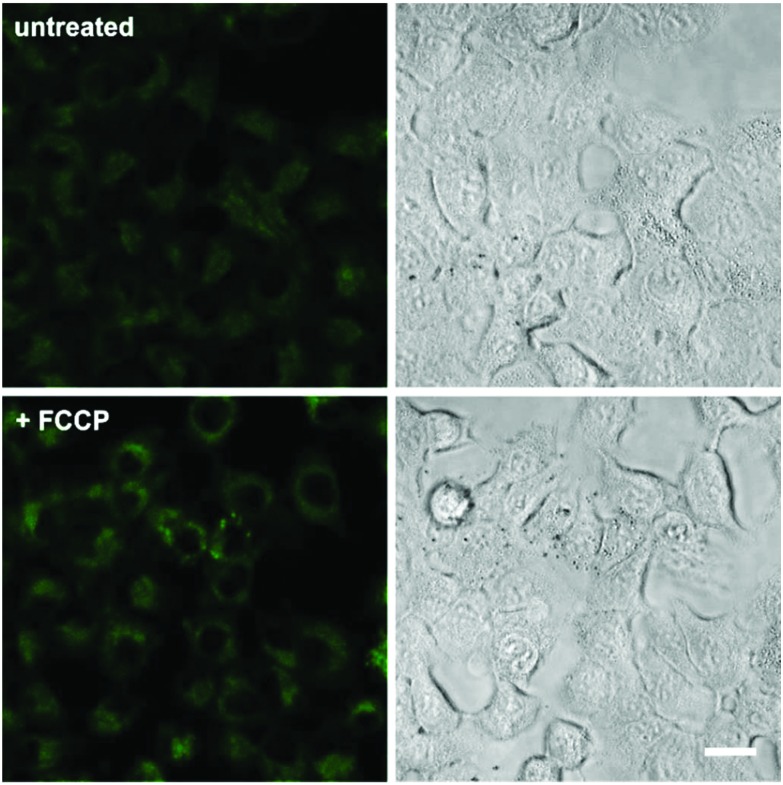
Fluorescence confocal microscopy images of A549 cells upon incubation with compound **4**. Cells were pre-incubated or not with 1 μM FCCP, and then treated with compound **4** (3 μM) for 15 min. Fluorescence images are shown in the left panels and brightfield images on the right. Quantitative analysis of the fluorescence intensity in both conditions is shown in Fig. S8.† Scale bar: 10 μm.

In view of the excellent properties of compound **4** as a Mg^2+^-activatable fluorescent probe, we examined its capabilities for cell imaging using A549 human adenocarcinoma cells. A549 cells were incubated or not with carbonyl cyanide *p*-(trifluoromethoxy)phenylhydrazone (FCCP), a molecule that acts as a mitochondria uncoupler and leads to an increase in Mg^2+^ concentration resulting from ATP hydrolysis and/or Mg^2+^ efflux from mitochondria.^19^,^32^ We acquired fluorescence confocal microscopy images of both untreated and FCCP-treated cells that had been incubated with the same concentration of compound **4**, and observed brighter intracellular emission in the latter due to significantly higher intracellular Mg^2+^ levels (Fig. 4 and Fig. S8 in ESI† for quantitative image analysis). We also examined the intracellular localisation of compound **4** by co-staining with different subcellular markers (*i.e.* LysoTracker Red and MitoTracker Red). Dual-colour images suggested that compound **4** mainly localised in the cytoplasm of A549 cells, with slightly preferential accumulation in the mitochondria over lysosomal compartments (Fig. S9 in ESI†). Furthermore, we performed cell viability experiments and confirmed that the incubation of A549 cells with compound **4** did not induce any significant cytotoxicity at the concentrations used for *in vitro* characterisation and cell imaging (Fig. S10 in ESI†). Altogether, these experiments assert the value of compound **4** as a fluorescent activatable probe for the detection of physiological Mg^2+^ concentrations, both *in vitro* and in cells.

**Fig. 5:**
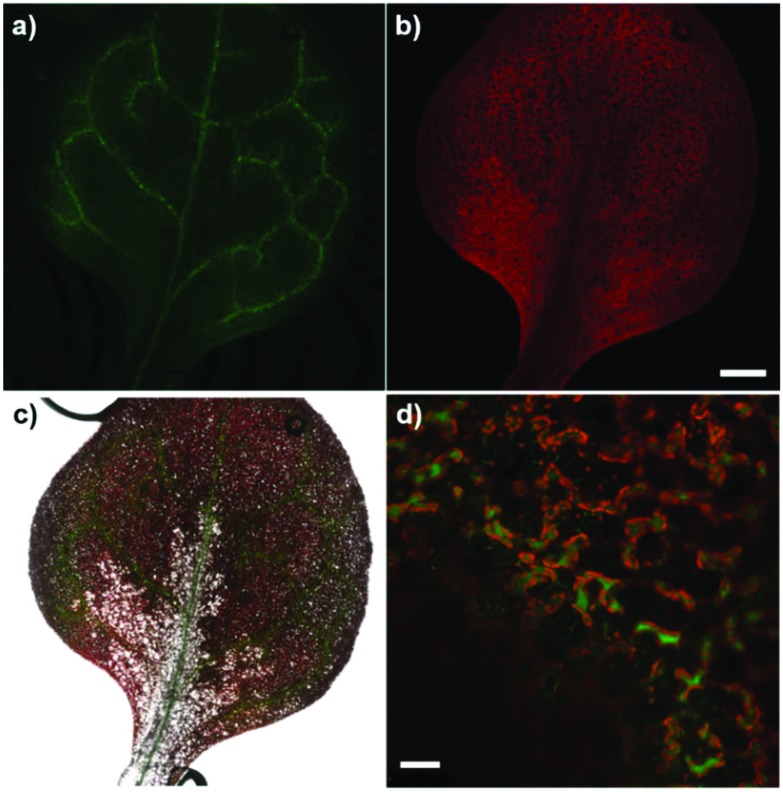
Fluorescence confocal microscopy images of *Arabidopsis thaliana* after treatment with compound **4**. Fluorescence images highlight the accumulation of compound **4** in the vasculature of new leaves upon cotyledon loading: (a) green fluorescence emission from compound **4**, (b) red fluorescence emission from chlorophyll, (c) merged fluorescent images from (a) and (b) including an overlay brightfield image. Scale bar: 250 μm. (d) High-magnification dual-colour image highlighting the localisation of compound **4** in chlorophyll-rich cells in the vascular region. Scale bar: 25 μm.

Finally, to examine the versatility of compound **4** for imaging in complex biological systems, we tested it in the plant model *Arabidopsis thaliana*, where Mg^2+^ ions accumulate around chlorophyll biomolecules in the chloroplasts. We applied compound **4** to the cotyledons and detected bright green fluorescence in the vasculature of new leaves (Fig. 5). Furthermore, high-magnification fluorescence images indicated the preferential localisation of compound **4** in chlorophyll-rich cells, such as mesophyll cells in the vascular regions (Fig. 5d).

## Conclusions

In summary, we developed a novel BODIPY activatable fluorophore for the detection and imaging of physiological concentrations of Mg^2+^ and high selectivity over other divalent cations, such as Ca^2+^. The combined lack of rotational freedom and effective quinolizine-based Mg^2+^ chelation function as the activation mechanisms for enhanced fluorescence amplification, enabling the imaging of free intracellular Mg^2+^ ions in live cells and tissues.

## Experimental section

### General methods

Commercially available reagents were used without further purification. Thin-layer chromatography was conducted on Merck silica gel 60 F254 sheets and visualized by UV (254 and 365 nm). Silica gel (particle size 35–70 μm) was used for column chromatography. ^1^H and ^13^C spectra were recorded in a Bruker Avance 500 spectrometer (at 500 and 125 MHz, respectively). Data for ^1^H NMR spectra reported as chemical shift *δ* (ppm), multiplicity, coupling constant (Hz) and integration. Data for ^13^C NMR spectra reported as chemical shifts relative to the solvent peak. HPLC-MS analysis was performed on a Waters Alliance 2695 separation module connected to a Waters PDA2996 photodiode array detector and a ZQ Micromass mass spectrometer (ESI-MS) with a Phenomenex® column (C_18_, 5 μm, 4.6 × 150 mm). Compounds were purified using a Waters semi-preparative HPLC system using a Phenomenex® column (C_18_ Axial, 10 μm, 21.2 × 150 mm) and UV detection.

### Synthesis of ethyl 1-formyl-4-oxo-4*H*-quinolizine-3-carboxylic acid (**1**)

Ethyl 1-formyl-4-oxo-4*H*-quinolizine-3-carboxylate (1.04 g, 0.1 mmol, 1 eq.) was dissolved in a mixture of THF (40 mL) and EtOH (40 mL). 1 M aqueous NaOH (5 mL, 1.3 eq.) was added and the reaction was left to stir for 2 h at r.t. Removal of the solvents under vacuum rendered the product (1.11 g) in quantitative yield. ^1^H NMR (500 MHz, D_2_O) *δ* 9.86 (d, *J* = 1.1 Hz, 1H), 9.37 (ddt, *J* = 7.3, 1.5, 0.8 Hz, 1H), 9.19 (dd, *J* = 9.0, 0.8 Hz, 1H), 8.58 (d, *J* = 0.9 Hz, 1H), 8.13–8.08 (m, 1H), 7.59 (tt, *J* = 7.1, 1.2 Hz, 1H) ppm. ^13^C NMR (125 MHz, D_2_O) *δ* 191.1, 172.9, 157.4, 146.0, 138.7, 130.0, 122.4, 119.6, 109.4 ppm.

### Synthesis and characterisation of BODIPY derivatives (**2–4**)

Compounds **2**, **3** and **4** were synthesized using modified reported procedures^30^,^31^ using the aldehyde **1** as the starting material. For the synthesis of compound **2**, compound **1** (150 mg, 0.63 mmol, 1 eq.) was dissolved in CH_2_Cl_2_ (50 mL), and pyrrole (437 μL, 6.3 mmol, 10 eq.) was then added followed by 1 drop of TFA. The reaction was left to stir overnight under N_2_. Additional pyrrole (437 μL, 6.3 mmol, 10 eq.) was then added followed by 1 more drop of TFA and the reaction was stirred under N_2_ for further 7 h. Afterwards, DDQ (143 mg, 0.63 mmol, 1 eq.) in CH_2_Cl_2_ (10 mL) was added dropwise and the reaction was left to stir for 20 min. Triethylamine (2.2 mL, 15.75 mmol, 25 eq.) followed by 48% BF_3_OEt_2_ in Et_2_O (1.2 mL, 9.45 mmol, 15 eq.) were added and the reaction was stirred for 3 h at r.t. under N_2_. Volatiles were removed under vacuum and flash column chromatography (CH_2_Cl_2_/MeOH, 92 : 8) followed by semi-preparative HPLC (0–100% ACN/H_2_O + 0.1% FA over 25 min) yielded compound **2** (2 mg) as an orange solid. ^1^H NMR (500 MHz, MeOD) *δ* 9.54 (d, *J* = 7.8 Hz, 1H), 8.53–6.37 (m, 1H), 8.03 (d, *J* = 10.1 Hz, 1H), 8.01–7.85 (m, 2H), 7.80 (s, 1H), 7.63 (dt, *J* = 13.5, 6.3 Hz, 2H), 6.95 (t, *J* = 5.0 Hz, 1H), 6.71–6.52 (m, 2H) ppm. ^13^C NMR (125 MHz, MeOD) *δ* 167.6, 160.2, 146.0, 145.7, 145.4, 140.0, 136.9, 136.3, 130.1, 129.7, 129.2, 124.3, 118.9, 118.5, 118.3 ppm. HRMS (ESI–) (*m*/*z*): [M – H]^–^ calcd for C_19_H_11_BF_2_N_3_O_3_ 378.0861; found 378.0841.

For the synthesis of compound **3**, compound **1** (150 mg, 0.63 mmol, 1 eq.) was dissolved in CH_2_Cl_2_ (50 mL), and 2-methylpyrrole (525 μL, 6.3 mmol, 10 eq.) was then added followed by 1 drop of TFA. The reaction was left to stir overnight under N_2_. Additional 2-methylpyrrole (263 μL, 3.15 mmol, 5 eq.) was then added followed by 1 more drop of TFA and the reaction was stirred under N_2_ for further 7 h. Afterwards, DDQ (143 mg, 0.63 mmol, 1 eq.) in CH_2_Cl_2_ (10 mL) was added dropwise and the reaction was left to stir for 20 min. Triethylamine (2.2 mL, 15.75 mmol, 25 eq.) followed by 48% BF_3_OEt_2_ in Et_2_O (1.2 mL, 9.45 mmol, 15 eq.) were added and the reaction was stirred for 3 h at r.t. under N_2_. Volatiles were removed under vacuum and flash column chromatography (CH_2_Cl_2_ : MeOH, 96 : 4) followed by semi-preparative HPLC (0–100% ACN/H_2_O + 0.1% FA over 25 min) yielded compound **3** (17 mg) as an orange solid. ^1^H NMR (500 MHz, CD_2_Cl_2_) *δ* 9.48 (d, *J* = 7.3 Hz, 1H), 8.63 (s, 1H), 7.90 (d, *J* = 8.9 Hz, 1H), 7.82–7.73 (m, 1H), 7.56–7.47 (m, 1H), 6.59 (d, *J* = 4.1 Hz, 2H), 6.32 (d, *J* = 4.2 Hz, 2H), 2.68 (s, 6H) ppm. ^13^C NMR (125 MHz, CD_2_Cl_2_) *δ* 165.4, 160.1, 159.6, 144.8, 141.5, 135.3, 134.6, 129.3, 128.9, 125.6, 125.1, 124.7, 120.3, 118.9, 110.0, 14.7 ppm. HRMS (ESI+) (*m*/*z*): [M]^+^ calcd for C_21_H_16_BF_2_N_3_O_3_ 407.1253; found 407.1251.

For the synthesis of compound **4**, compound **1** (26 mg, 0.1 mmol, 1 eq.) was dissolved in CH_2_Cl_2_ (5 mL), and 2,4-dimethylpyrrole (20 μL, 0.2 mmol, 2 eq.) was then added followed by 1 drop of TFA. The reaction was left to stir overnight under N_2_. Afterwards, DDQ (23 mg, 0.1 mmol, 1 eq.) in CH_2_Cl_2_ (5 mL) was added dropwise and the reaction was left to stir for 20 min. Triethylamine (350 μL, 2.5 mmol, 25 eq.) followed by 48% BF_3_OEt_2_ in diethyl ether (185 μL, 1.5 mmol, 15 eq.) were added and the reaction was stirred for 3 h at r.t. under N_2_. Volatiles were removed under vacuum and flash column chromatography (CH_2_Cl_2_ : MeOH, 95 : 5) followed by semi-preparative HPLC (0–100% ACN/H_2_O + 0.1% FA over 25 min) yielded compound **4** (13 mg) as an orange solid in a 25% yield over the 3 steps. ^1^H NMR (500 MHz, CDCl_3_) *δ* 9.50 (t, *J* = 8.8 Hz, 1H), 8.58 (d, *J* = 8.1 Hz, 1H), 7.88–7.76 (m, 2H), 7.55–7.45 (m, 1H), 6.04 (s, 2H), 2.62 (s, 6H), 1.38 (s, 6H) ppm. ^13^C NMR (125 MHz, CDCl_3_) *δ* 165.6, 160.0, 159.7, 157.6, 144.8, 143.7, 142.0, 141.0, 135.8, 133.1, 131.9, 129.2, 123.8, 122.1, 118.7, 110.9, 14.9, 14.7 ppm. HRMS (ESI+) (*m*/*z*): [M + H]^+^ calcd for C_23_H_21_BF_2_N_3_O_3_ 436.1644; found 436.1643.

### *In vitro* spectral characterisation

Spectroscopic and quantum yield data were recorded on a Synergy HT spectrophotometer (Biotek). Compounds were dissolved at the indicated concentrations and spectra were recorded at r.t. Spectra are represented as means from at least two independent experiments with *n* = 3. When applicable, quantum yields were calculated by measuring the integrated emission area of the fluorescence spectra and comparing it to the area measured for fluorescein in basic ethanol as reference.^41^

### Fluorescence imaging in A549 cells

Human lung A549 adenocarcinoma cells (ATCC CCL-185) were grown using Dulbecco's Modified Eagle Medium (DMEM) supplemented with 10% fetal bovine serum (FBS), antibiotics (100 U mL^–1^ penicillin and 100 mg mL^–1^ streptomycin) and 2 mM l-glutamine in a humidified atmosphere at 37 °C with 5% CO_2_. A549 cells were regularly passaged in T-75 cell culture flasks. For microscopy experiments, cells were plated on glass chamber slides Lab-Tek™ II (Nunc) the day before imaging, reaching 75% to 90% confluence on the day of the experiment. For imaging experiments, cells were incubated for 15 min at 37 °C with compound **4**, washed once with PBS and imaged under a Zeiss LSM 510 META fluorescence confocal microscope equipped with a live cell imaging stage. Fluorescence and brightfield images were acquired using a 40× oil objectives. Fluorescent probes were excited with 488 nm (for compound **4**) or 543 nm (for MitoTracker Red and LysoTracker Red) lasers. Confocal microscopy images were analysed and processed with ImageJ.

### Fluorescence imaging in *Arabidopsis*

*Arabidopsis thaliana* seeds of the Col-0 ecotype were surface sterilised using 10% (v/v) bleach for 10 min, followed by one wash in 70% EtOH, and 5 rinses in sterile ddH_2_O. Seeds were then plated individually on 0.5× Murashige-Skoog media, solidified with 2% (w/v) Phytoagar. Seeds were stratified at 4 °C for 3 days before being transferred to a vertical position in a controlled growth chamber under long day conditions (16 h light: 8 h dark), with 100 μmol m^–2^ s^–1^ white light and a constant temperature of 21 °C. At 11 days after germination, plates were placed flat to encourage the cotyledons to grow away from the media. On day 12, the cotyledons were treated with 0.3 μL of 2.5% (v/v) Adigor for 1 h to improve cotyledon permeability. A 0.3 μL drop of 10 mM compound **4** was then applied to each cotyledon and the seedlings were incubated for around 4 h. The true leaves were then carefully removed, avoiding any contact with the probe, and mounted on glass slides using ddH_2_O. The leaves were imaged using Leica 5× HC PL Fluotar or 20× HCX APO w objectives on a Leica TCS SP8 Confocal. Samples were excited at 488 nm and emission collected at 500–540 nm for compound **4** and 650–705 nm for chlorophyll.

## Conflicts of interest

There are no conflicts to declare.

## Supplementary Material

Supplementary information
