# Supplementary material for: A fluorescent activatable probe for imaging intracellular Mg2+
Source: Org Biomol Chem. 2017 Dec 19;16(2):239–44. doi: 10.1039/c7ob02965a (PMC5789582; doi:10.1039/c7ob02965a)
Supplement: Supplementary file 1 [file OB-016-C7OB02965A-s001.pdf]

This journal is © The Royal Society of Chemistry 2017

Electronic Supplementary Information

A Fluorescent Activatable Probe for Imaging Intracellular Mg^{2+}

Ryan Treadwell, Fabio de Moliner, Ramon Subiros-Funosas, Toby Hurd, Kirsten Knox and Marc Vendrell

Table of Contents:

1. Synthetic procedures.
2. NMR spectra.
3. Additional spectroscopy and imaging data.

1. Synthetic procedures.

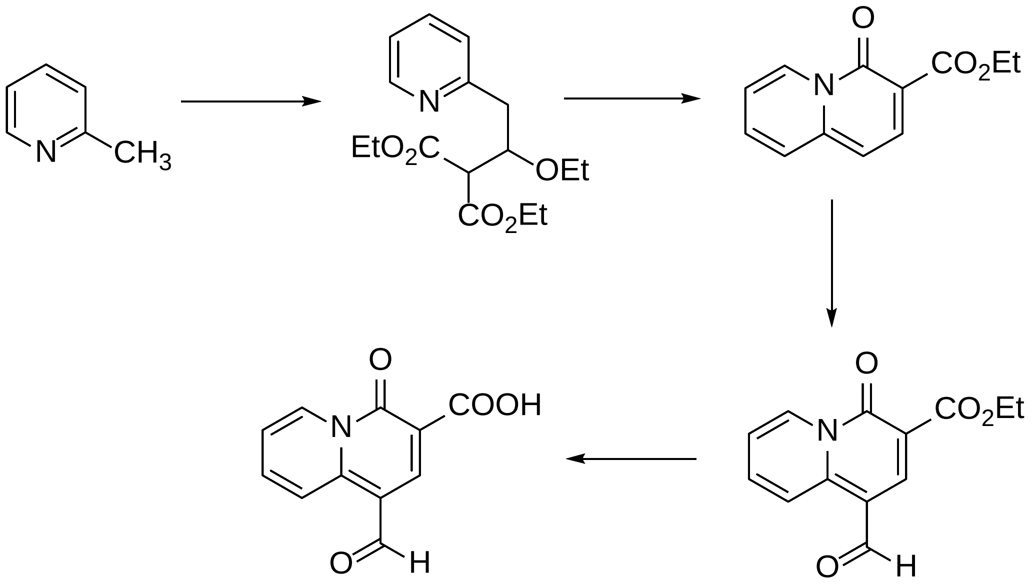

1

Scheme S1. Synthetic scheme for the preparation of the aldehyde **1**.

2-[1-ethoxy-2-(2-pyridinyl)ethyl]-1,3-diethyl ester

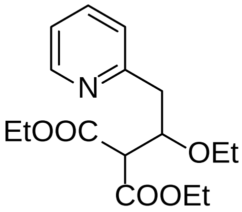

Picoline (5 g, 54 mmol, 1 eq.) was dissolved in dry THF (30 mL) and the resulting solution was slowly added under N_2 at –78°C to a 2.0 M solution of LDA in THF/heptane/ethylbenzene (33 mL, 66 mmol, 1.25 eq.). After stirring for 30 min, ethoxymethylmalonate (12.74 g, 5.9 mmol, 1.1 eq.) in dry THF (30 mL) was added over a 10-min period and the reaction was left to stir for further 20 min while allowing the temperature to increase to –20°C. After 3 h, the reaction was judged complete by TLC and HPLC-MS, poured into deionised H_2O (500 mL) and extracted using CH_2Cl_2 (3 × 300 mL). The combined organic layers were dried over $MgSO_4$ and concentrated *in vacuo*. Flash column chromatography (EtOAc/hexane, 1:1) yielded the product (8.64 g) as a dark orange oil in 52% yield.

^{1}H NMR (500 MHz, $CDCl_3$) δ 8.55 (ddd, *J* = 5.0, 1.8, 0.9 Hz, 1H), 7.62 (td, *J* = 7.7, 1.9 Hz, 1H), 7.24 (m, 1H), 7.15 (ddd, *J* = 7.5, 4.9, 1.2 Hz, 1H), 4.49 (m, 1H), 4.20 (dq, *J* = 8.4, 7.1 Hz, 4H), 3.63 (d, *J* = 8.0 Hz, 1H), 3.47 (dq, *J* = 9.1, 7.0 Hz, 2H), 3.31 (dq, *J* = 9.1, 7.0 Hz, 1H), 3.22 (m, 1H), 1.27 (td, *J* = 7.1, 3.8 Hz, 3H), 0.98 (t, *J* = 7.0 Hz, 6H) ppm.

^{13}C NMR (125 MHz, $CDCl_3$) δ 167.5, 167.3, 158.2, 148.9, 136.4, 124.6, 121.6, 66.4, 61.4, 61.4, 56.9, 41.4, 15.2, 14.1, 14.0 ppm.

Ethyl 4-*oxo*-4*H*-quinolizine-3-carboxylate

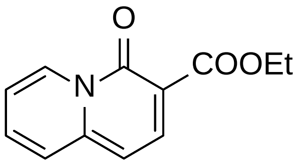

2-[1-ethoxy-2-(2-pyridinyl)ethyl]-1,3-diethyl ester (6 g, 27.62 mmol) was dissolved in xylene (60 mL) and heated under reflux for 48 h. Afterwards, the solvent was removed *in vacuo* and flash column chromatography (EtOAc/hexane, 1:1) yielded the product (1.06 g) as an orange crystalline solid in 25% yield.

^{1}H NMR (500 MHz, $CDCl_3$) δ 8.55 (ddd, *J* = 5.0, 1.8, 0.9 Hz, 1H), 7.62 (td, *J* = 7.7, 1.9 Hz, 1H), 7.26 (dt, *J* = 7.9, 1.1 Hz, 1H), 7.15 (ddd, *J* = 7.5, 4.9, 1.2 Hz, 1H), 4.48 – 4.42 (m, 1H), 4.20 (dq, *J* = 8.4, 7.1 Hz, 4H), 3.63 (d, *J* = 8.0 Hz, 1H), 3.47 (dq, *J* = 9.1, 7.0 Hz, 1H), 3.31 (dq, *J* = 9.1, 7.0 Hz, 1H), 3.22 – 3.07 (m, 2H), 1.27 (td, *J* = 7.1, 3.8 Hz, 6H), 0.98 (t, *J* = 7.0 Hz, 3H) ppm.

^{13}C NMR (125 MHz, $CDCl_3$) δ 166.2, 155.9, 146.1, 141.0, 133.5, 129.3, 125.4, 116.4, 106.9, 102.2, 60.9, 14.5 ppm.

Ethyl 1-formyl-4-*oxo*-4*H*-quinolizine-3-carboxylate

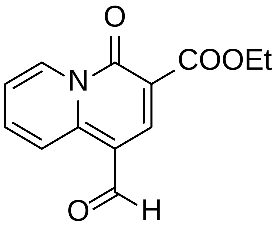

$POCl_3$ (1.35 mL, 14.5 mmol, 3 eq.) was added dropwise over 5 min to a solution of 2-[1-ethoxy-2-(2-pyridinyl)ethyl]-1,3-diethyl ester (1.05 g, 4.93 mmol, 1 eq.) in 2.5 mL of DMF and left to stir at r.t.. After 1 h, the reaction was judged complete by TLC, and the crude was diluted with H_2O (100 mL) and extracted using CH_2Cl_2 (3 x 100 mL). The combined organic layers were dried over $MgSO_4$ and concentrated under reduced pressure. Flash column chromatography (EtOAc/hexane, 4:1) yielded the product (1.04 g) as a yellow solid in 87% yield.

^{1}H NMR (500 MHz, $CDCl_3$) δ 9.93 (s, 1H), 9.61 (d, *J* = 1.3 Hz, 1H), 9.58 (m, 1H), 8.82 (s, 1H), 8.05 (m, 1H), 7.54 (td, *J* = 7.0, 1.4 Hz, 1H), 4.48 (q, *J* = 7.1 Hz, 2H), 1.47 (t, *J* = 7.1 Hz, 3H) ppm.

^{13}C NMR (125 MHz, $CDCl_3$) δ 188.2, 164.8, 149.0, 145.7, 139.2, 130.7, 123.9, 119.2, 108.8, 105.7, 61.4, 14.5 ppm.

2. NMR spectra

^{1}H-NMR

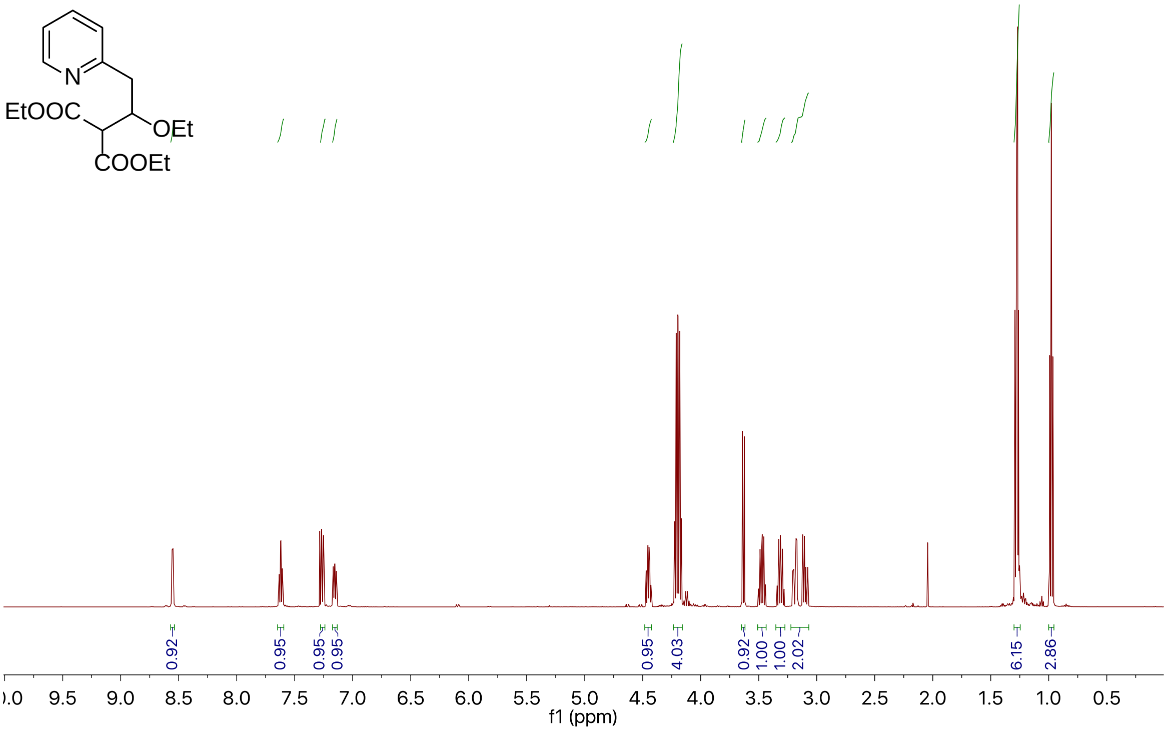

^{13}C-NMR

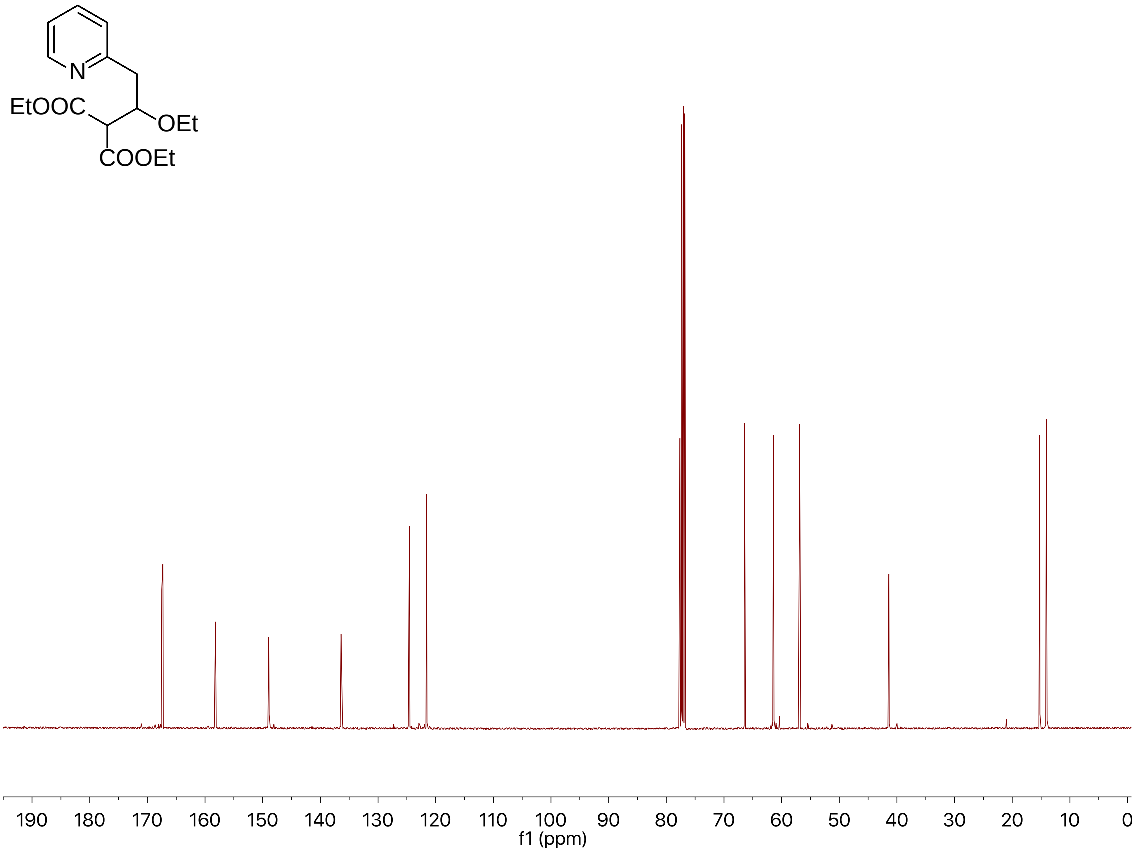

^{1}H-NMR

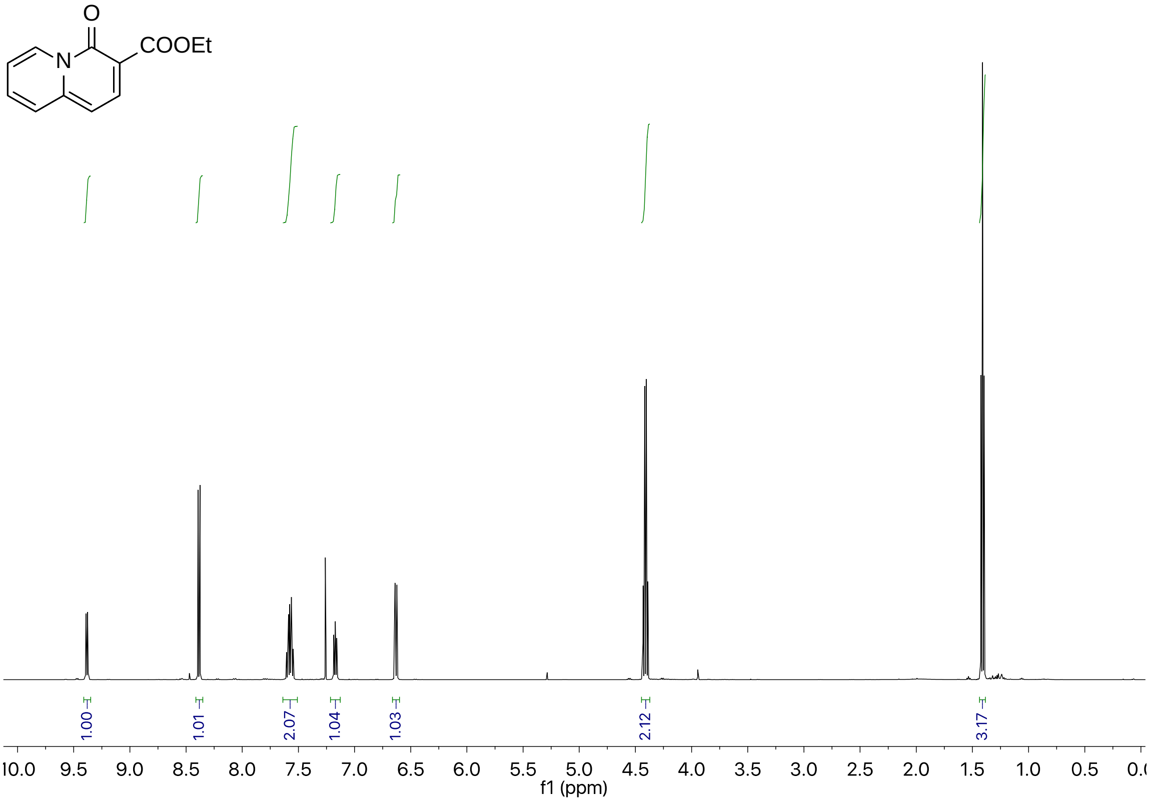

^{13}C-NMR

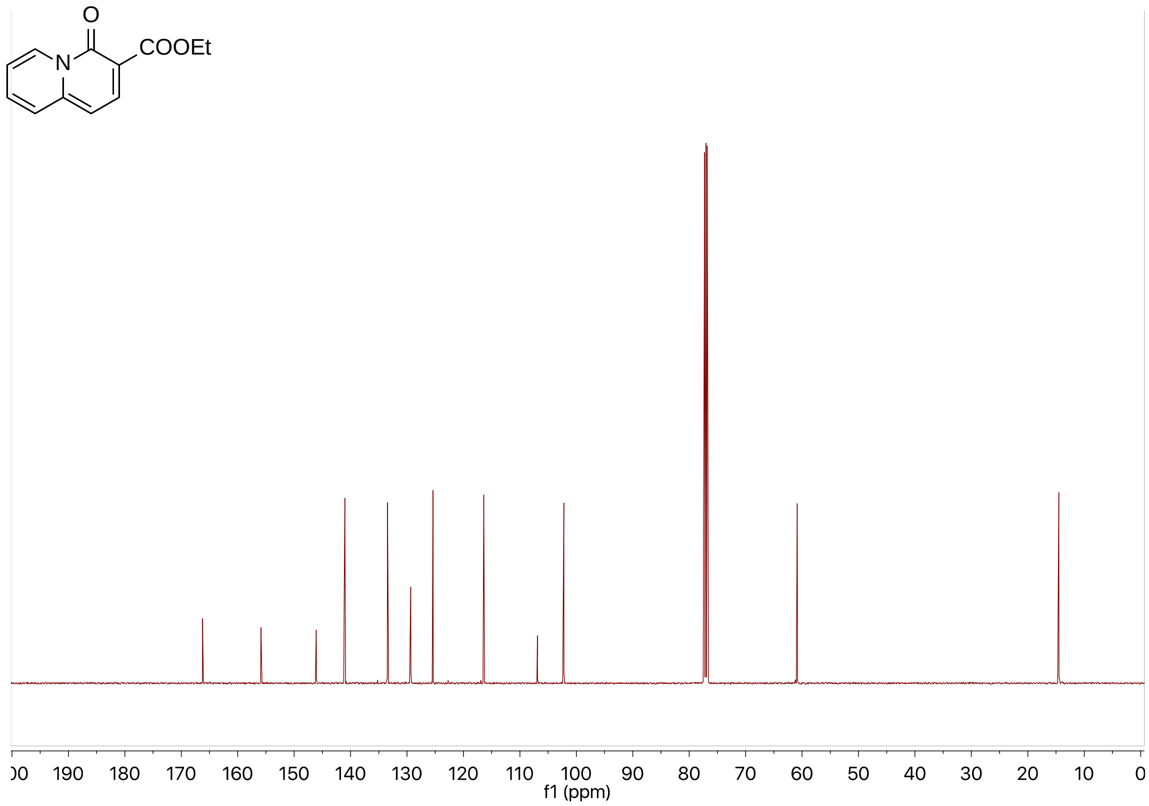

^{1}H-NMR

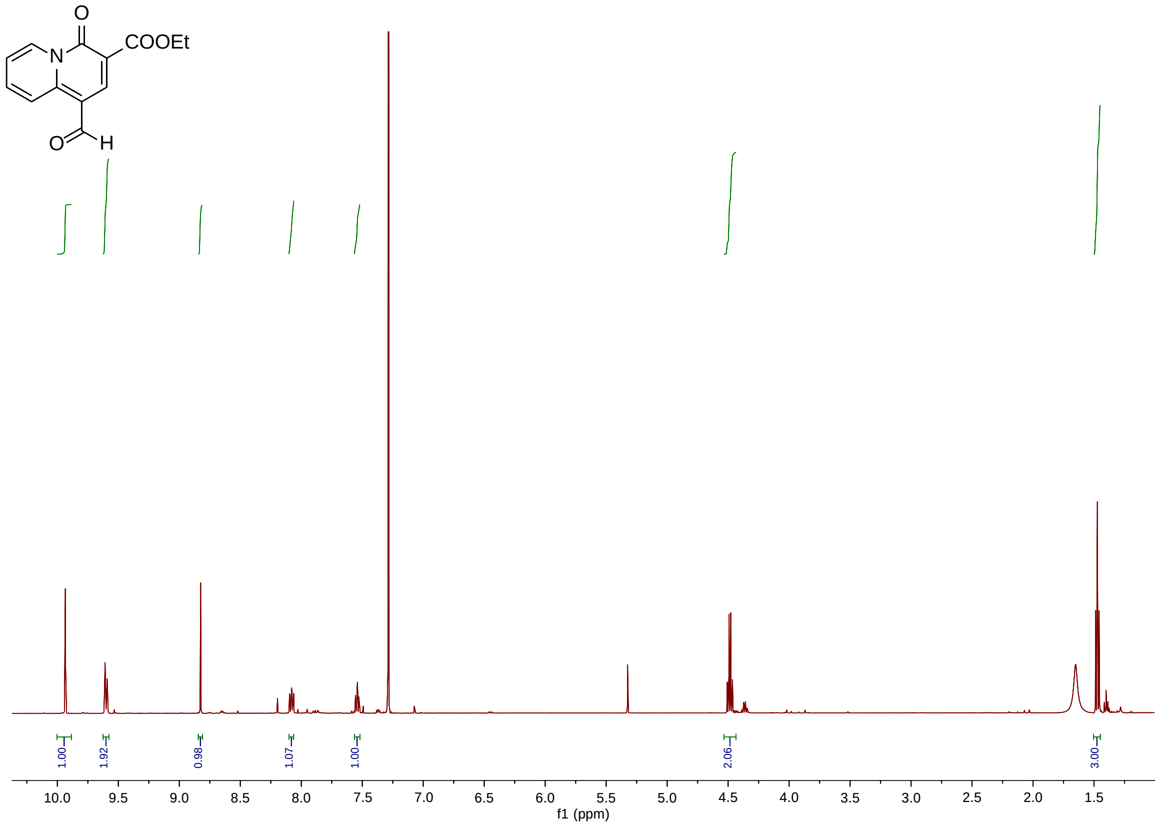

^{13}C-NMR

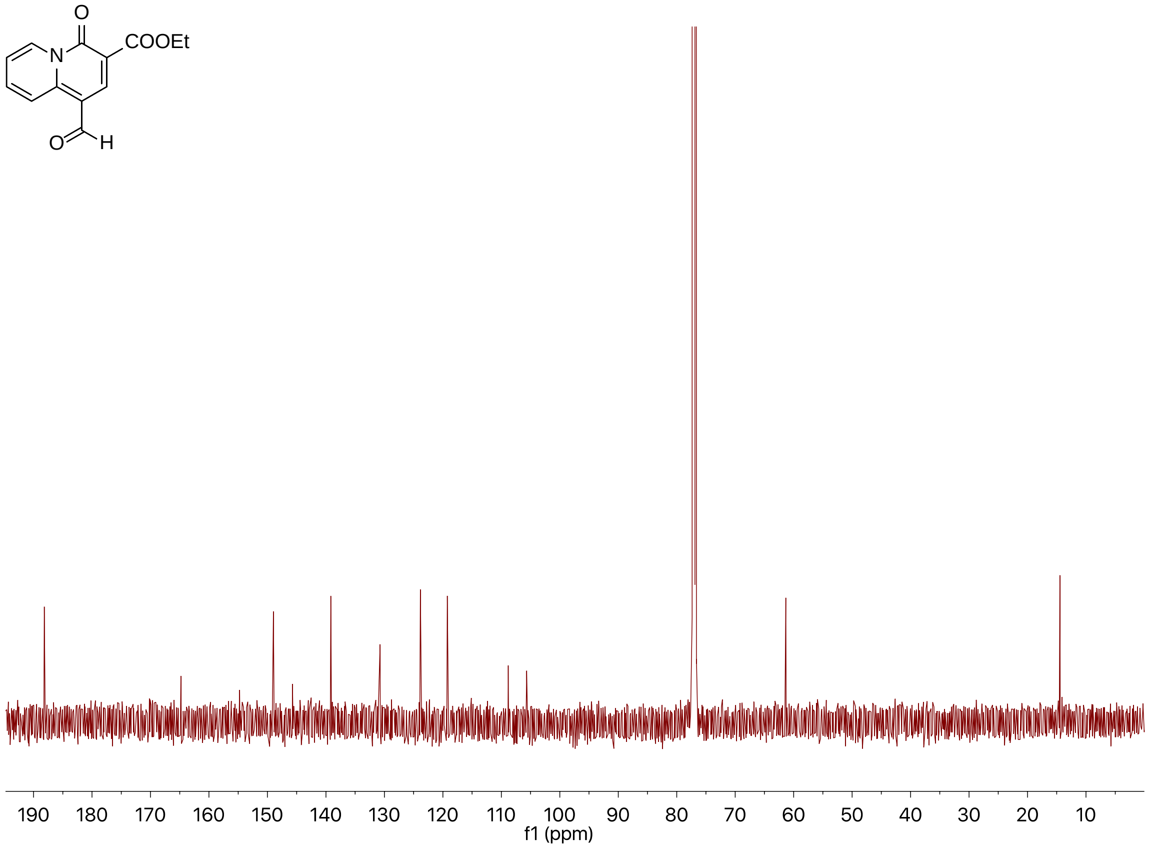

^{1}H-NMR (compound 1)

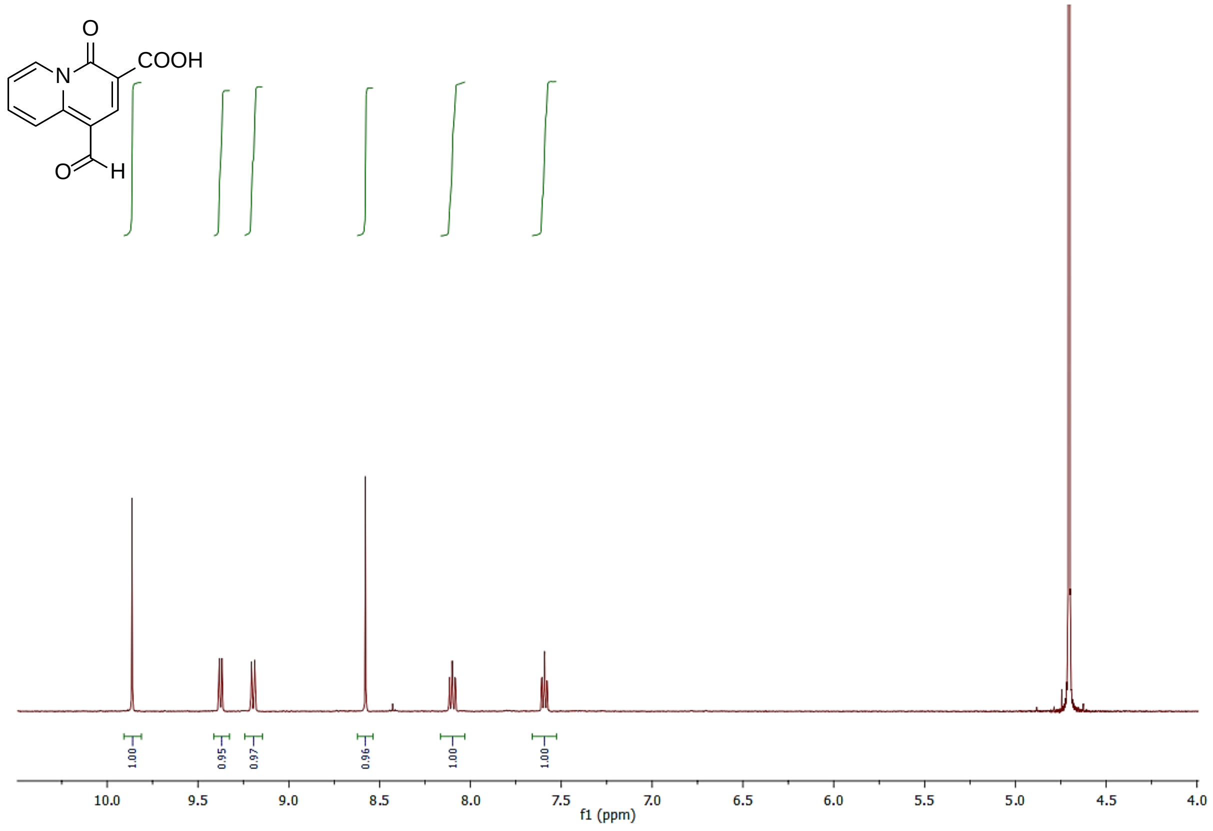

^{13}C-NMR (compound 1)

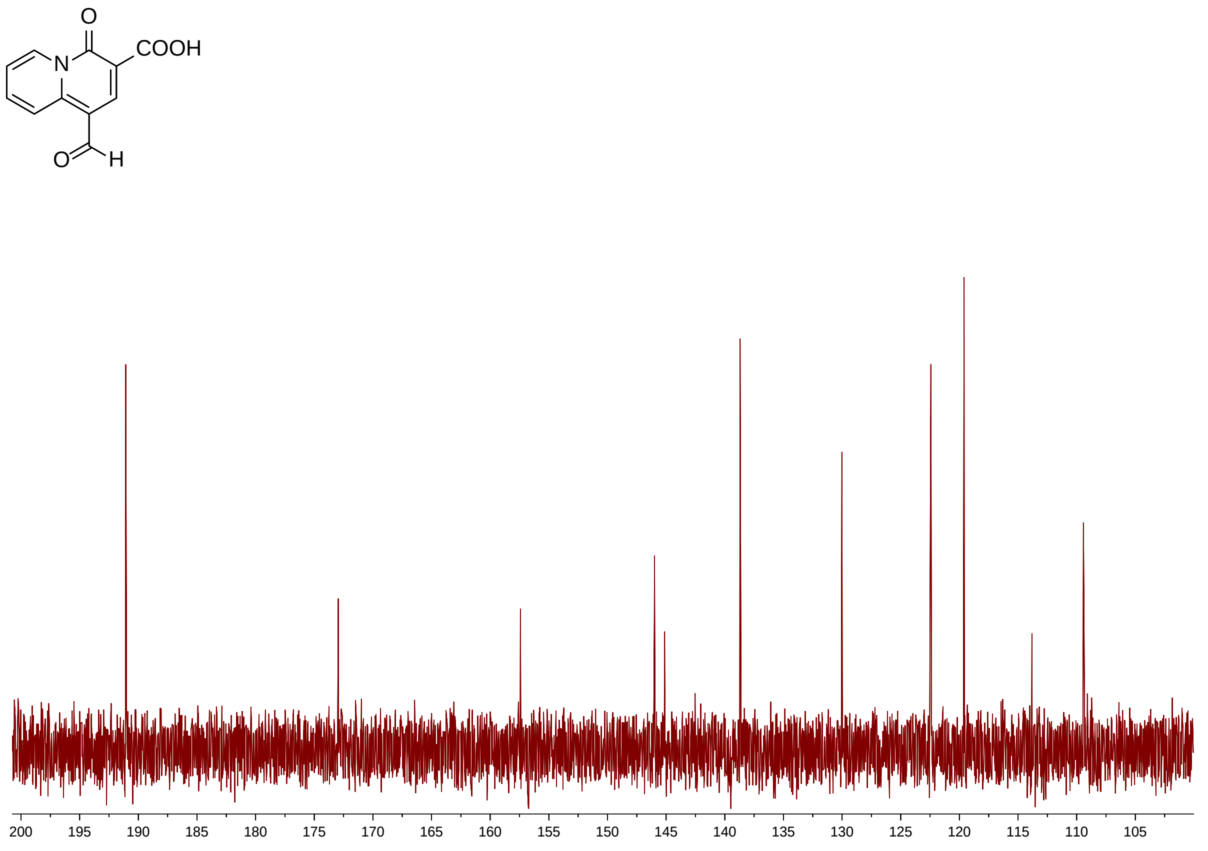

^{1}H-NMR (compound 2)

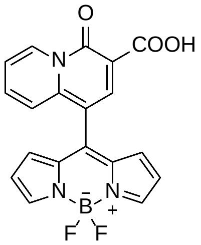

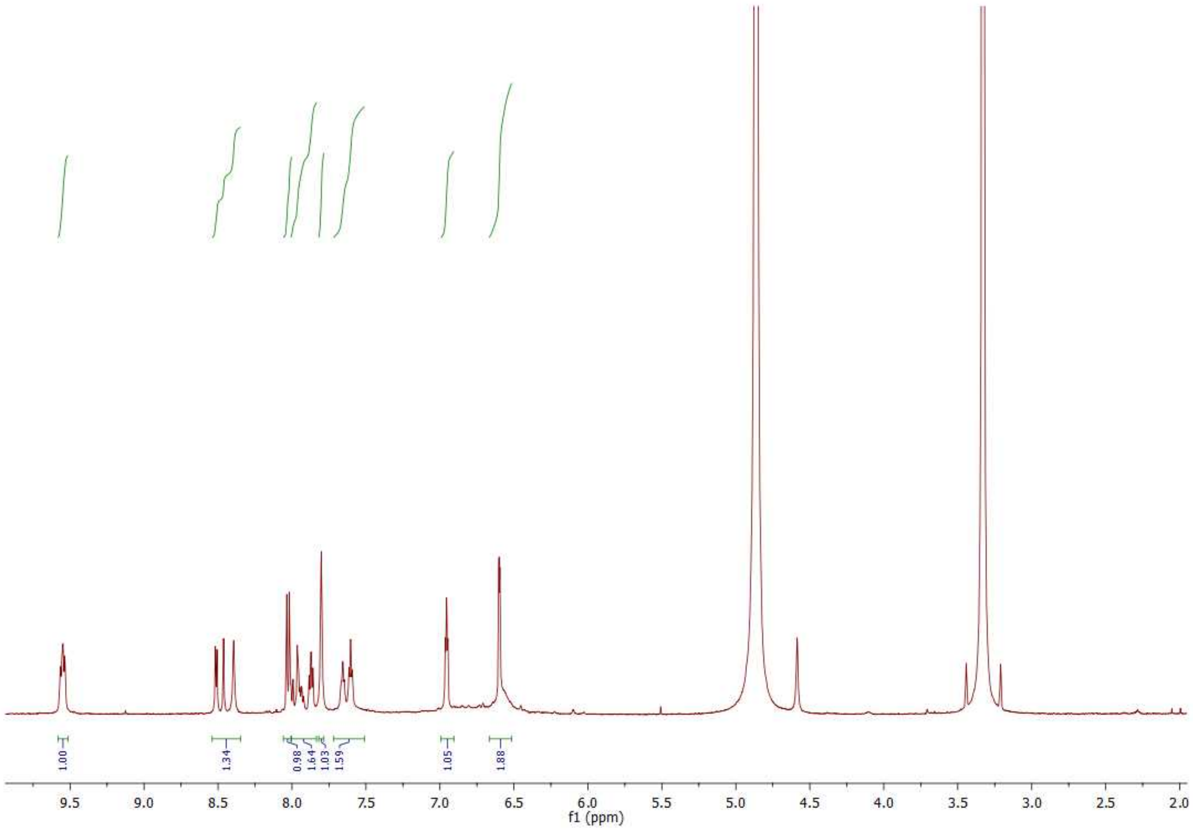

HSQC (compound 2)

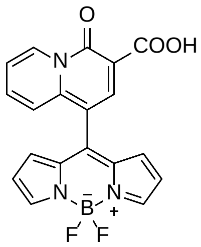

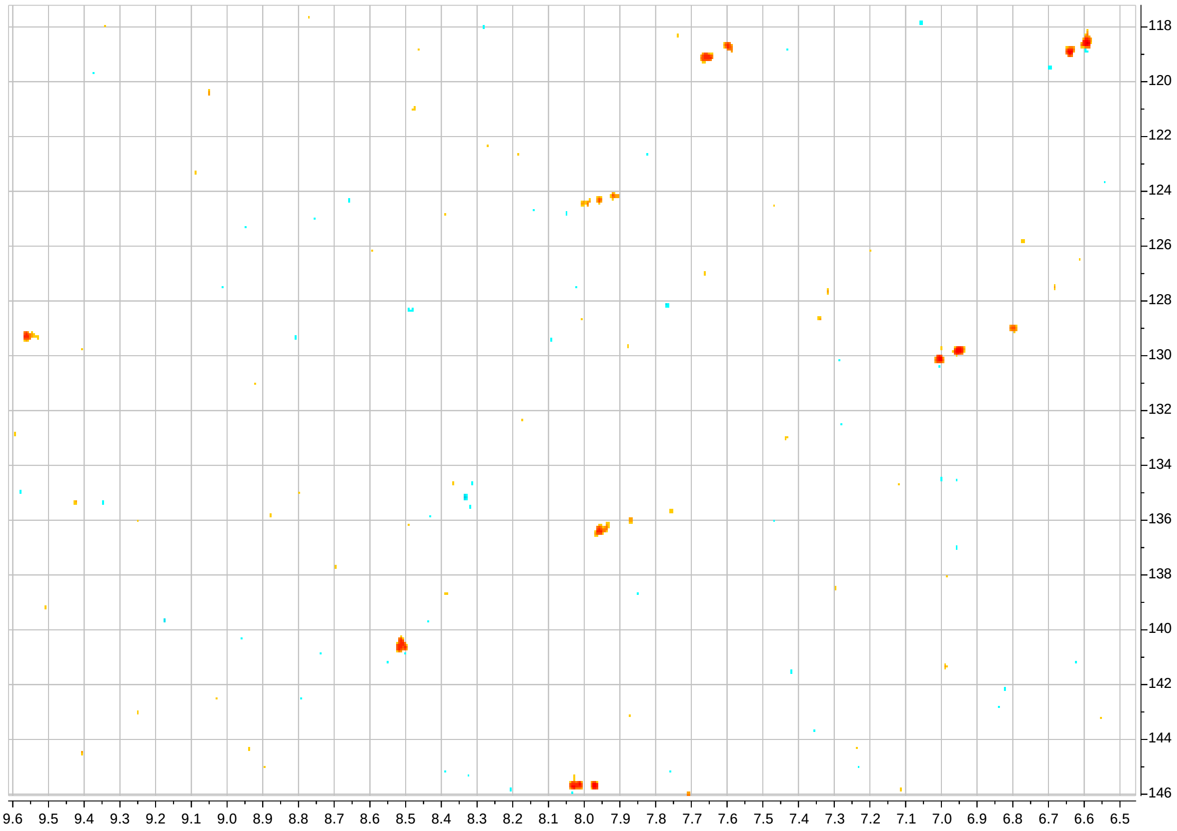

HMBC (compound 2)

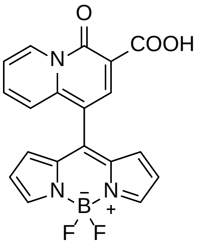

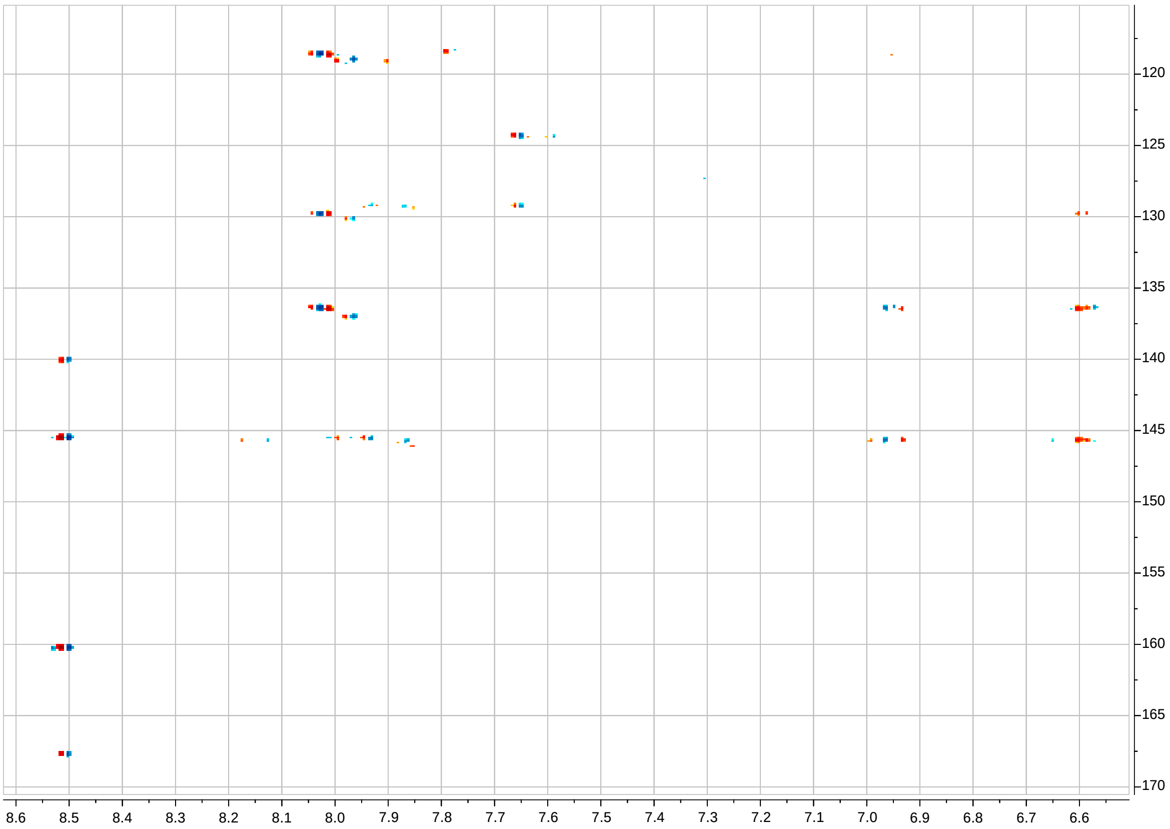

^{1}H-NMR (compound 3)

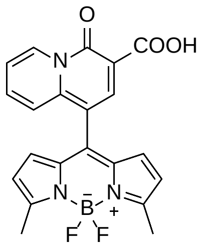

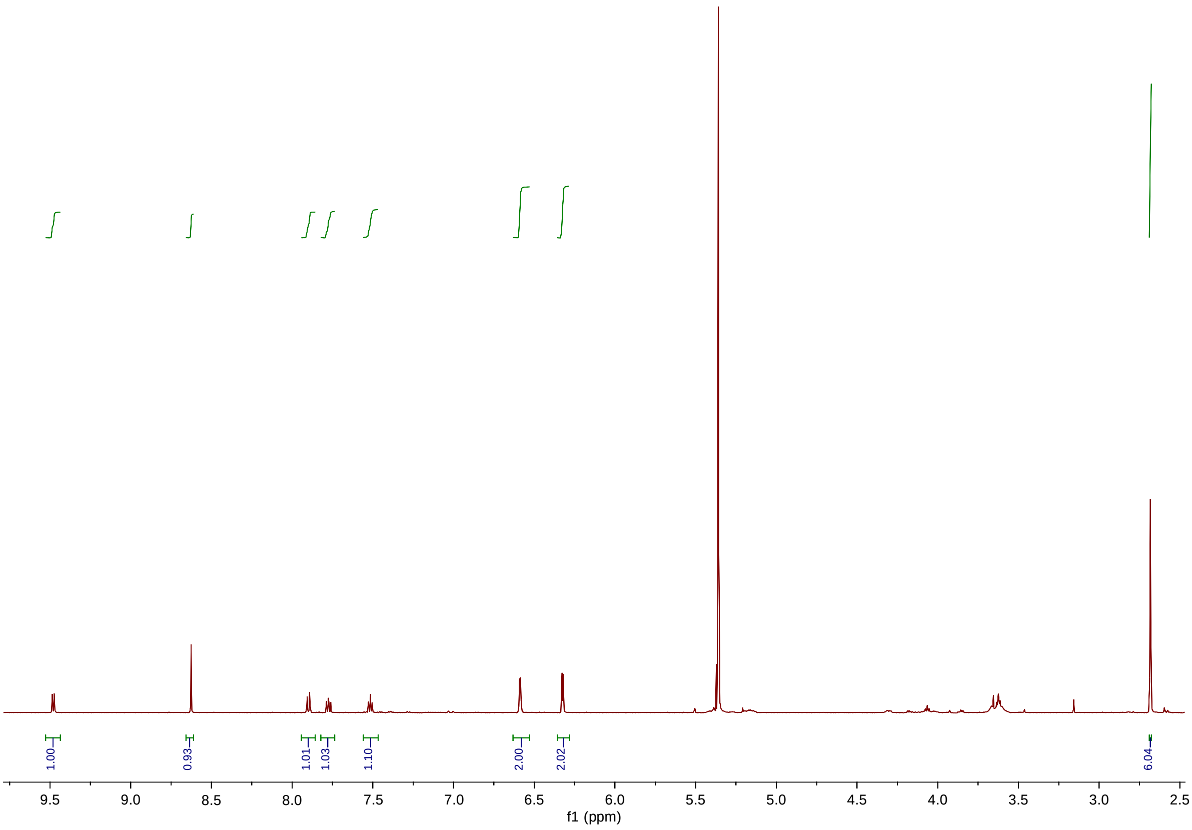

HSQC (compound 3)

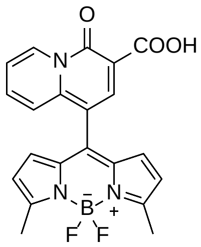

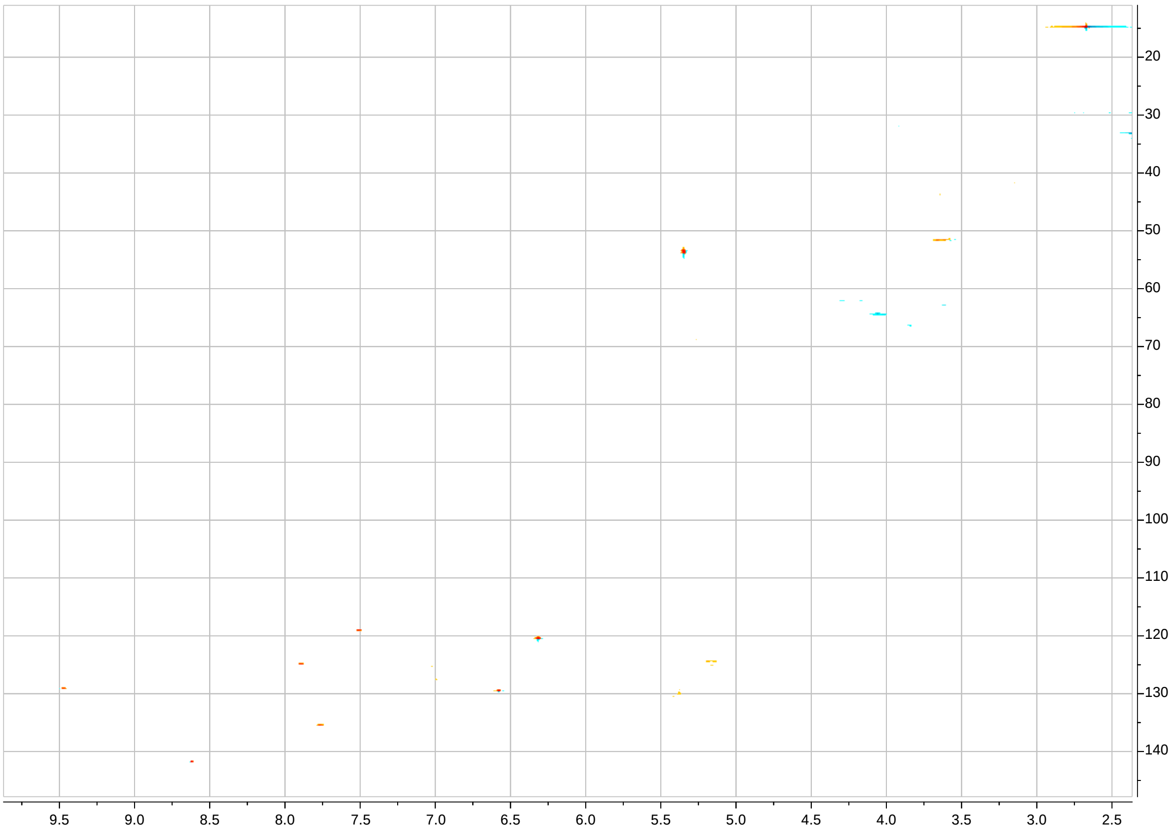

HSQC (expanded aromatic region) (compound 3)

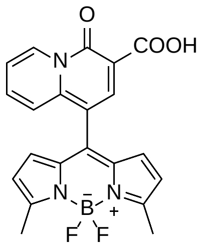

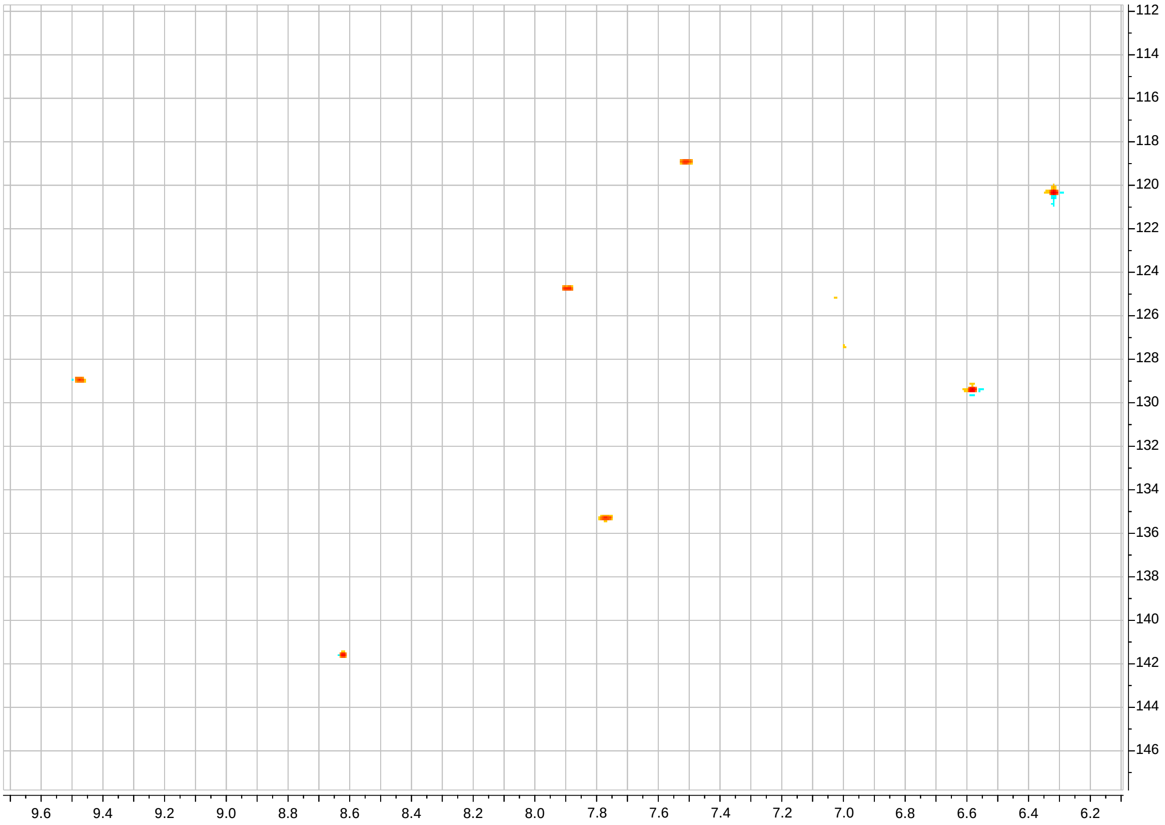

HMBC (compound 3)

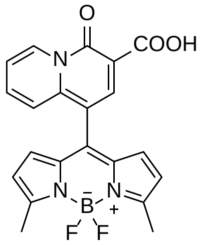

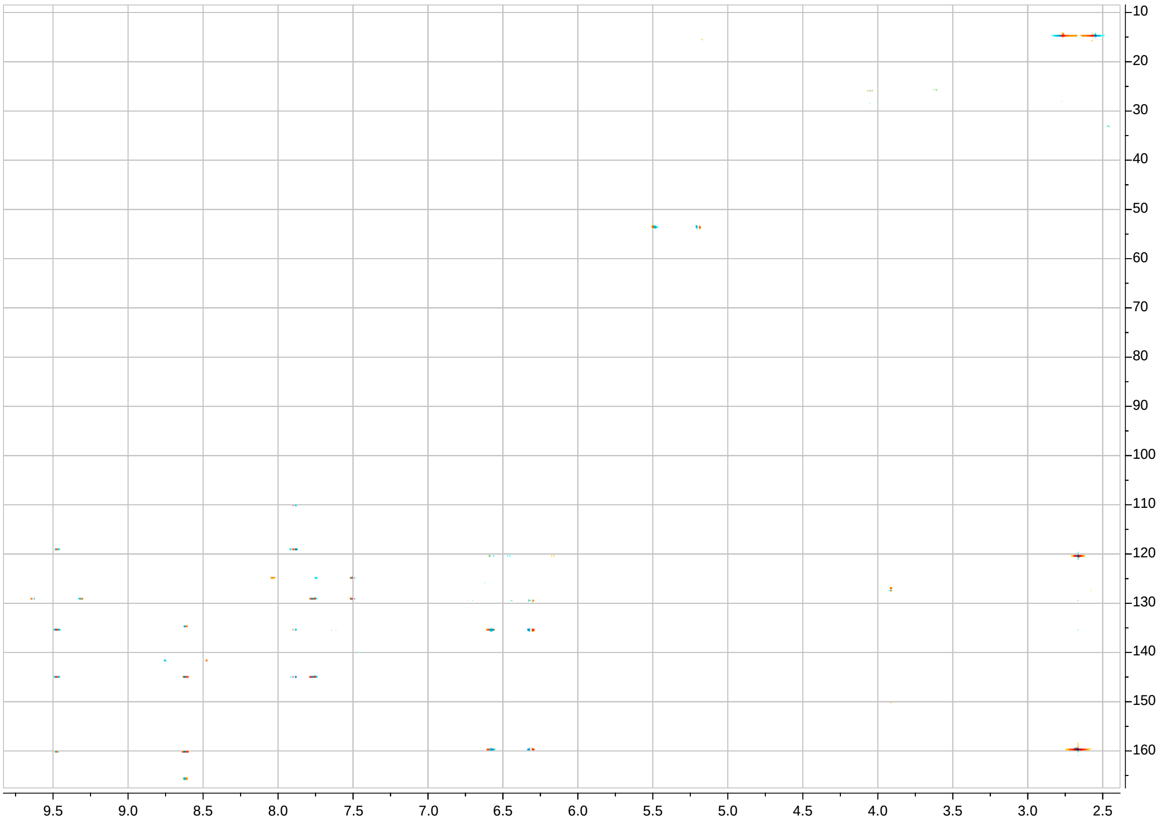

HMBC (expanded aromatic region) (compound 3)

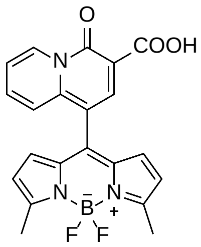

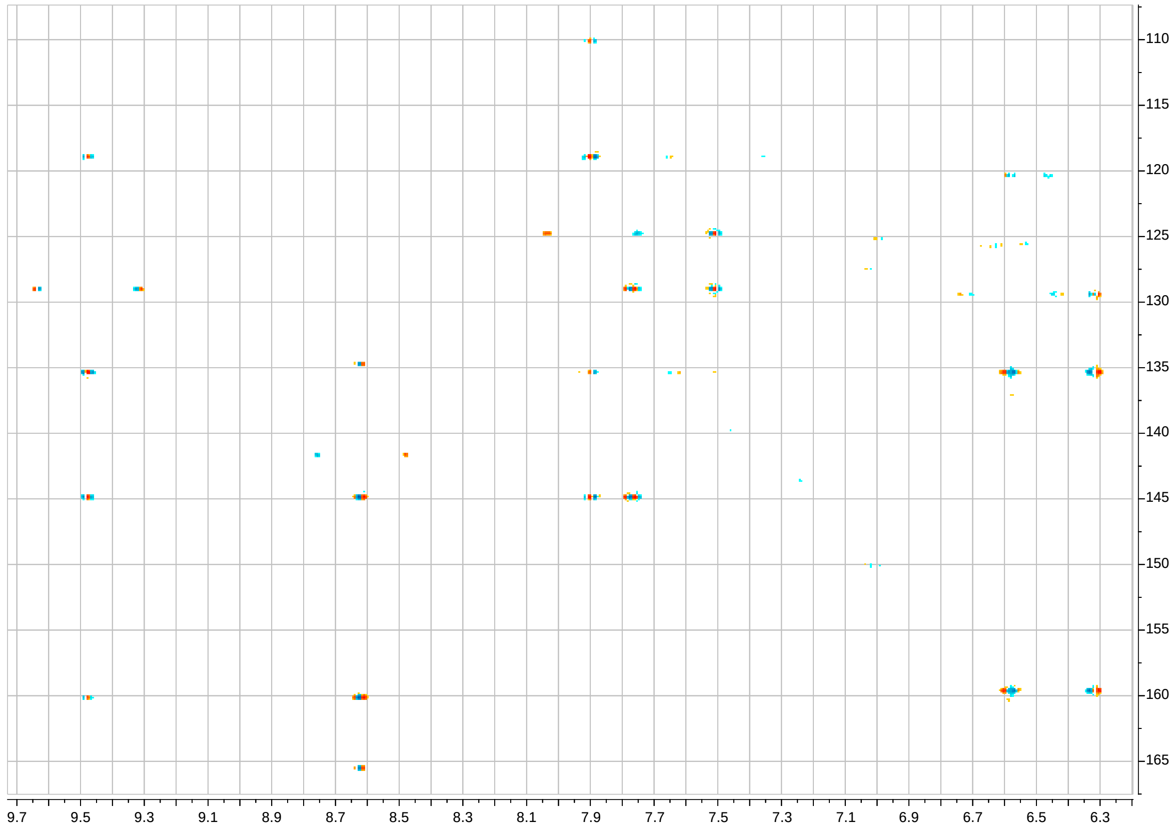

^{1}H-NMR (compound 4)

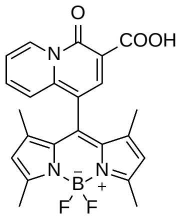

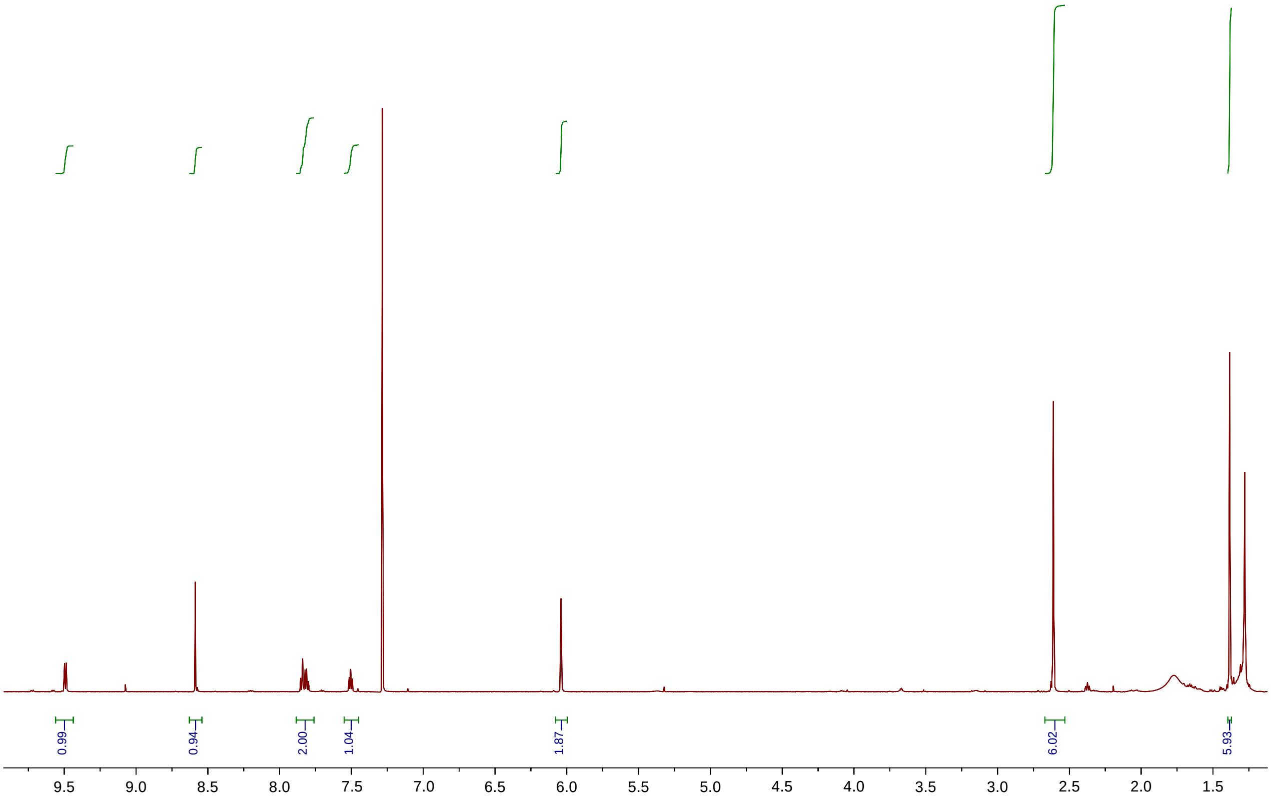

HSQC (compound 4)

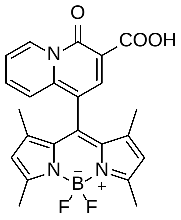

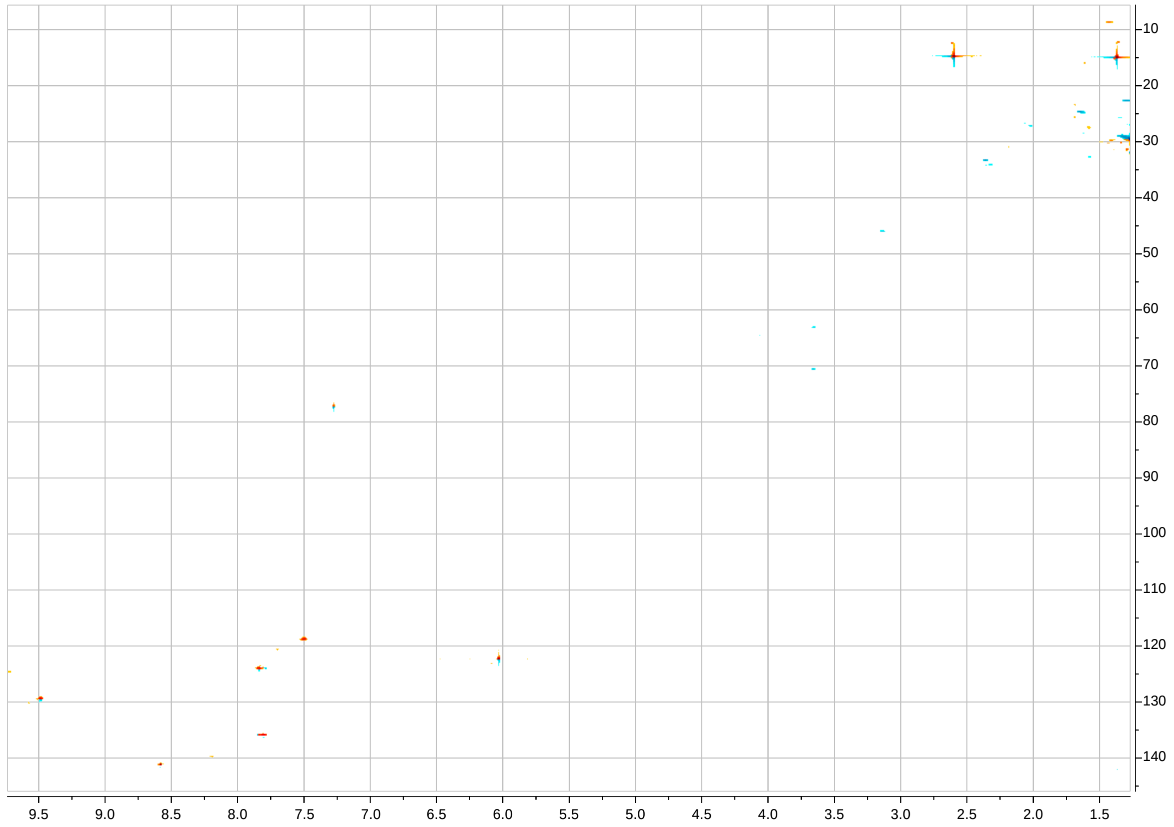

HSQC (expanded aromatic region) (compound 4)

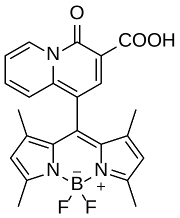

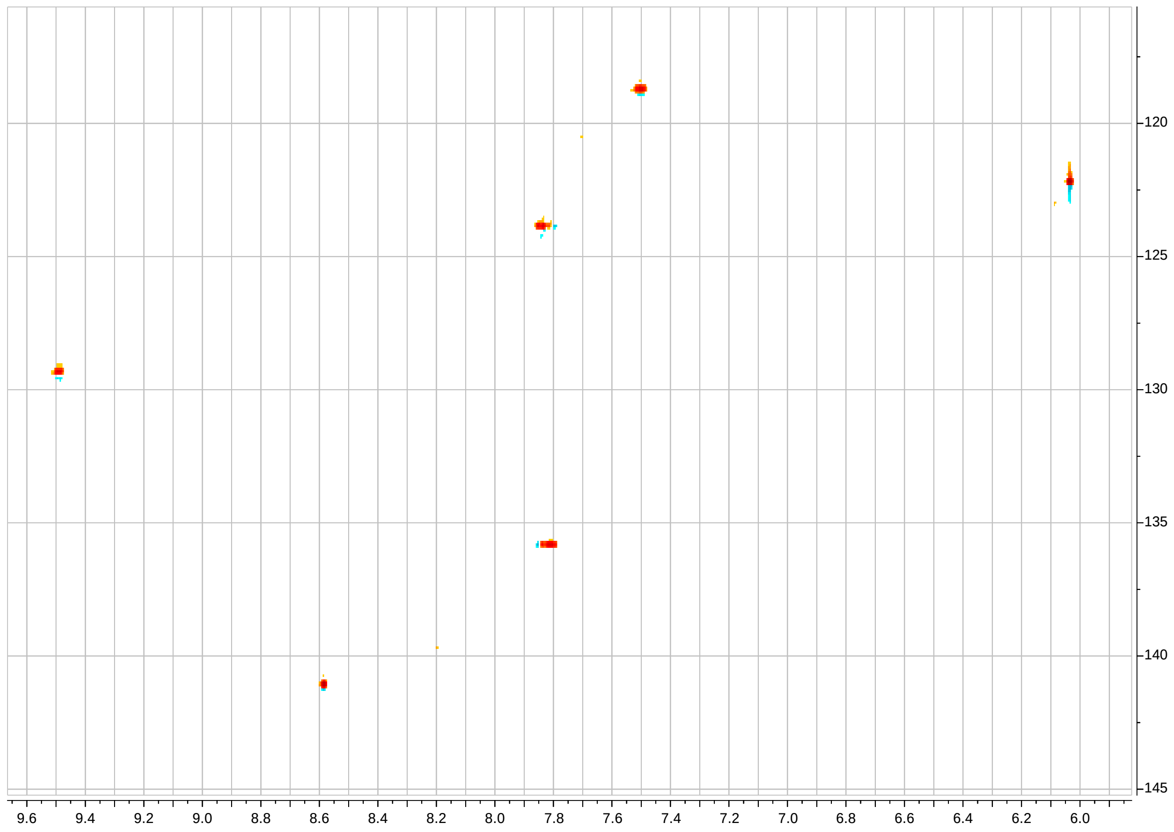

HMBC (compound 4)

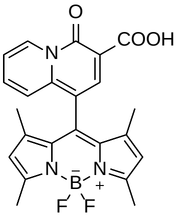

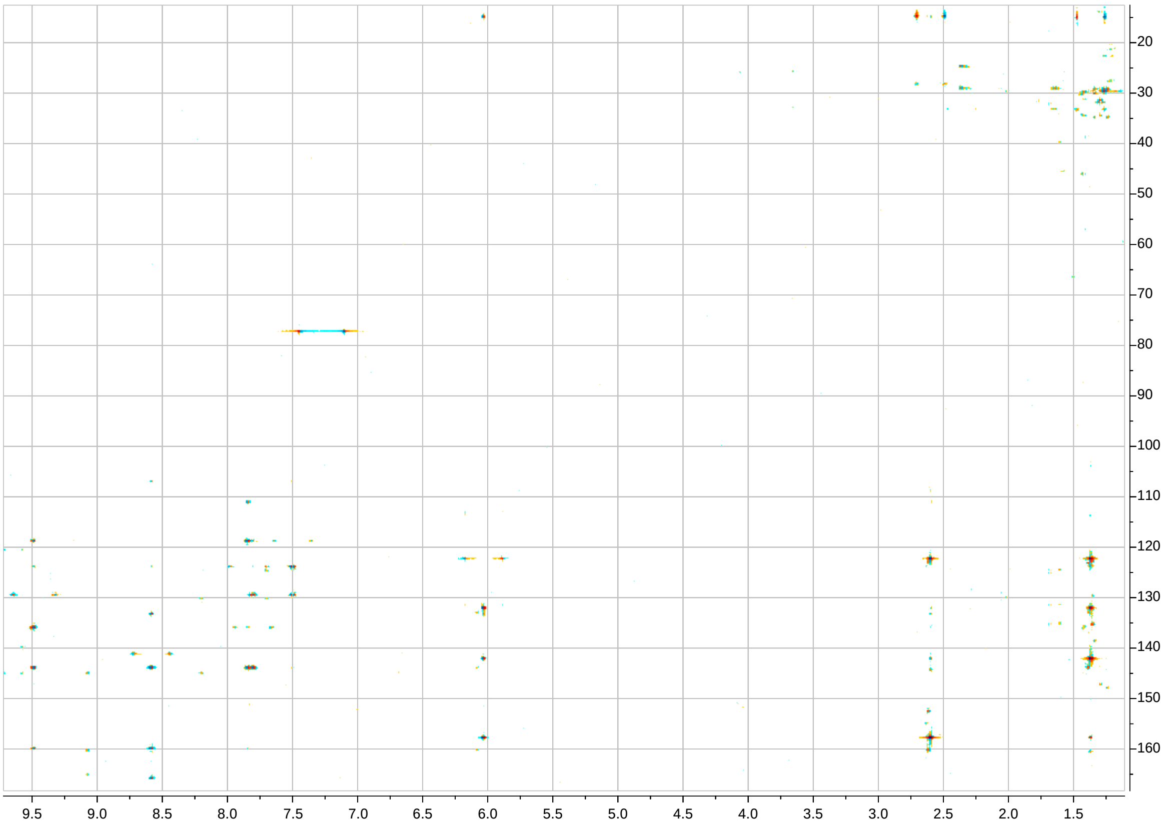

HMBC (expanded aromatic region) (compound 4)

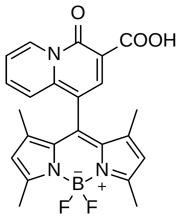

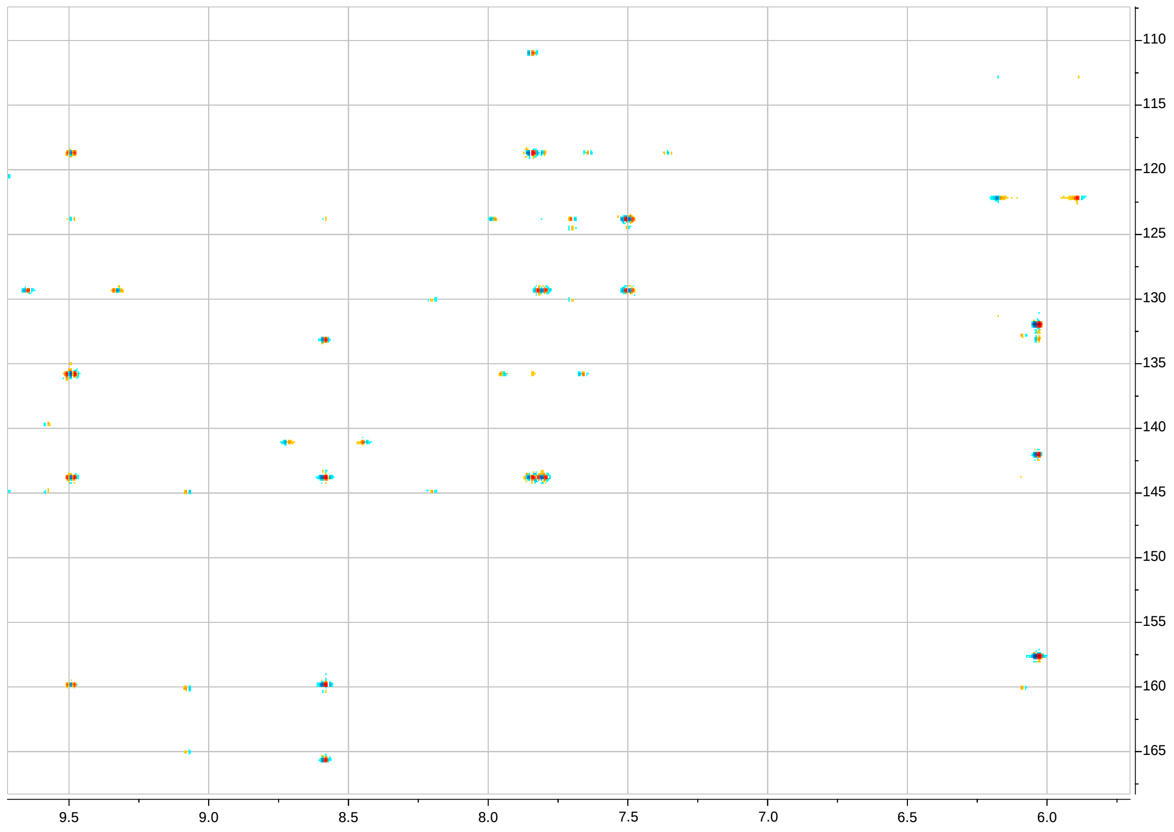

3. Additional spectroscopy and imaging data.

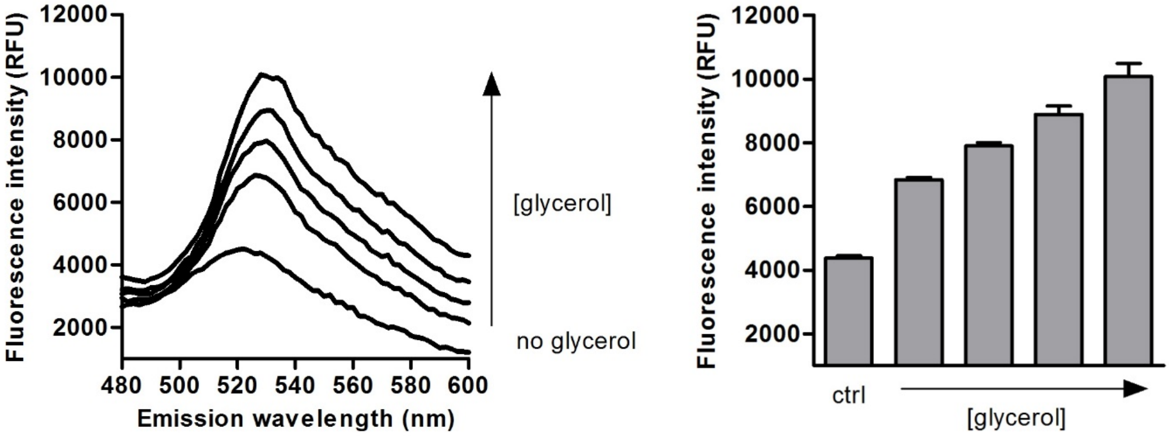

Figure S1. Fluorescence spectra ($\lambda_{exc.}$: 450 nm) of compound **2** upon incubation without and with increasing concentrations of glycerol.

	λ_{abs} (nm)	λ_{em} (nm)
**2**	506	520
**3**	500	520
**4**	508	520

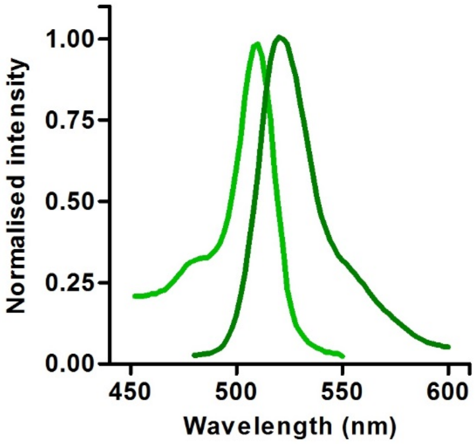

Figure S2. Spectral characterisation of the BODIPY compounds **2**-**4**. For compound **4**, absorbance (clear green line) and emission ($\lambda_{exc.}$: 450 nm, dark green line) spectra (10 μM) in EtOH.

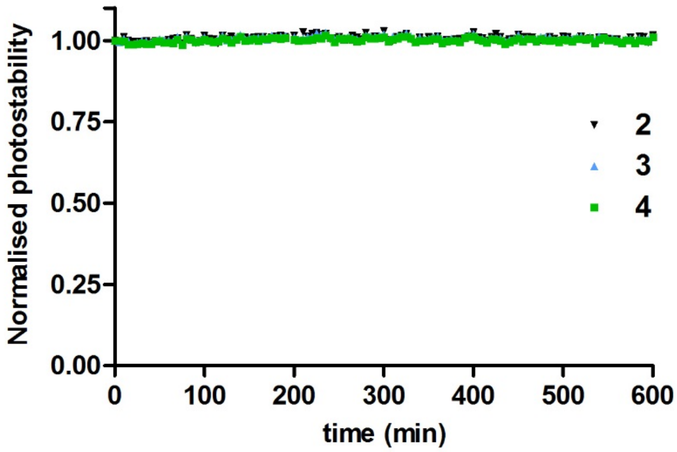

Figure S3. Photostability analysis of compounds **2**-**4** (100 μM) in PBS upon continuous light irradiation for 10 h.

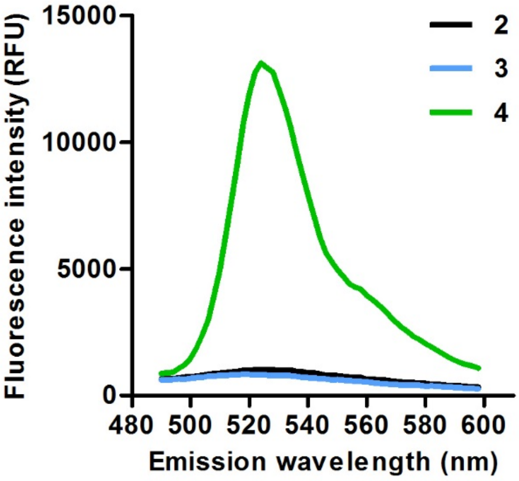

Figure S4. Fluorescence spectra ($\lambda_{exc.}$: 450 nm) of compounds **2**-**4** (20 μM) upon incubation with the same Mg^{2+}-containing solution (2 mM) and under the same experimental conditions.

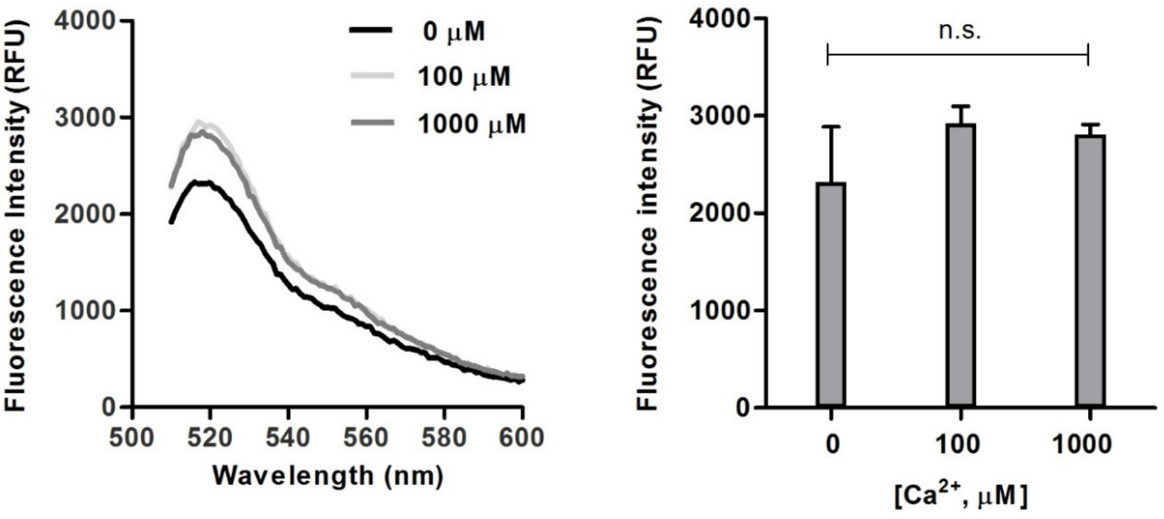

Figure S5. Fluorescence spectra ($\lambda_{exc.}$: 450 nm) and fluorescence intensity readouts of compound **4** upon incubation with low (100 μM) and high (1 mM) concentrations of Ca^{2+} within its physiological range. Values represented as means ± s.e.m (n = 3). n.s. for $p > 0.05$.

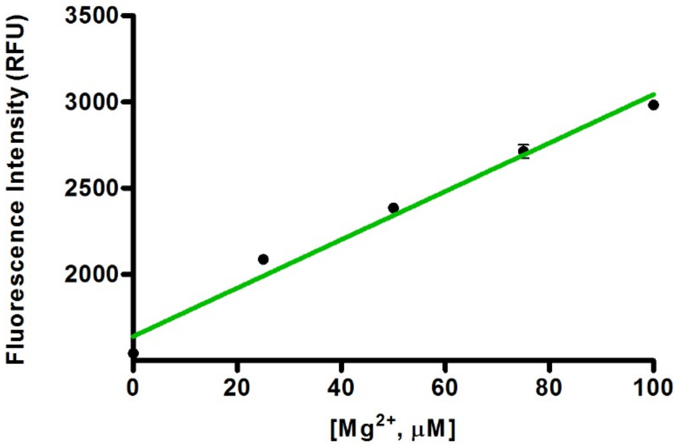

Figure S6. Determination of limit of detection of compound **4** for the detection of Mg^{2+}. Compound **4** (10 μM) was incubated with increasing concentrations of Mg^{2+} and the fluorescence emission was detected at r.t. Values represented as means ± s.e.m (n = 3), and the limit of detection was determined as 2 μM.

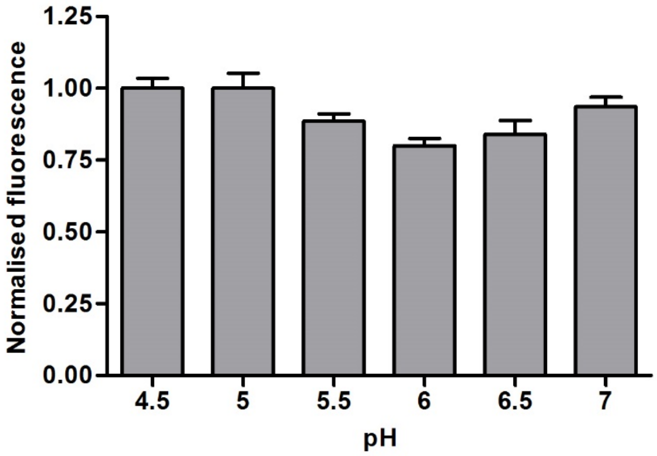

Figure S7. Normalised fluorescence intensity of compound **4** (10 μM) upon binding to Mg^{2+} (1 mM) in different buffers covering different pH values measured with ± 0.2 deviation. Values represented as means ± s.d (n = 3).

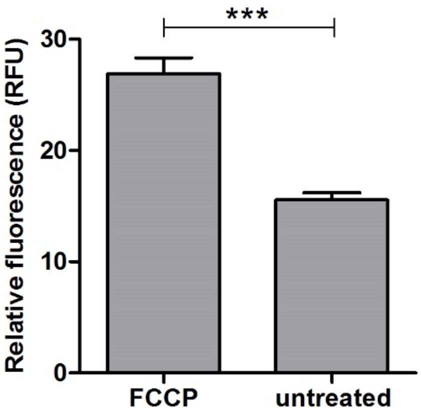

Figure S8. Fluorescence intensity quantification of FCCP-treated and untreated A549 cells after incubation with compound **4** (3 μM, 15 min). Image analysis was performed using Image J, including 10 regions of interest per condition covering a similar overall area. Values represented as means ± s.d. *** for $p < 0.001$.

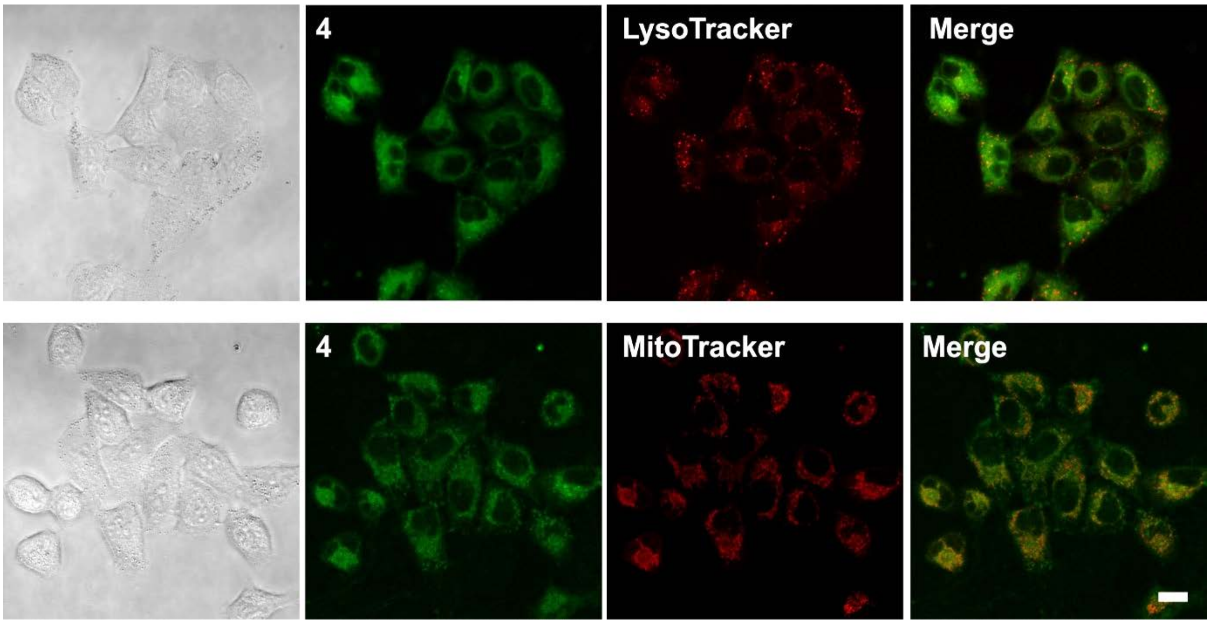

Figure S9. Confocal fluorescence microscopy images of A549 cells upon incubation with compound **4** and organelle-specific fluorescent markers. Cells were pre-incubated in culture media containing 5 mM $MgCl_2$ and then treated with compound **4** (3 μM) and the corresponding trackers for 15 min. Scale bar: 10 μm.

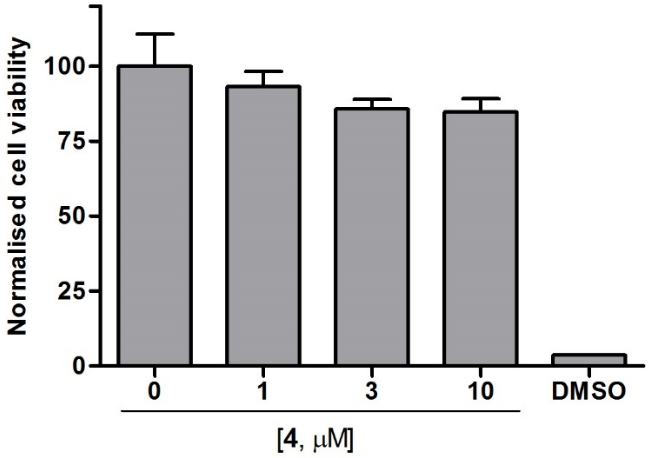

Figure S10. Cytotoxicity assays in A549 cells upon incubation with different concentrations of compound **4** and DMSO as a positive control. Cell viability was normalised to cells incubated under the same experimental conditions with only cell culture medium. Values represented as means ± s.d (n = 3).
